# Supplementary material for: Developing inhibitors of the guanosine triphosphate hydrolysis accelerating activity of Regulator of G protein Signaling-14[image]
Source: J Biol Chem. 2025 Aug 21;301(10):110611. doi: 10.1016/j.jbc.2025.110611 (PMC12495443; doi:10.1016/j.jbc.2025.110611)
Supplement: Supporting information [file mmc1.pdf]

Supplementary Information

Developing inhibitors of the guanosine triphosphate hydrolysis accelerating activity of Regulator of G protein Signaling-14

Percy S. Agogo-Mawuli [1], Isra Sadiya [2], Tigran M. Abramyan [3], Dustin E. Bosch [4], Kyle A. Emmitte [5], Luis M. Colón-Pérez [1], Mickey Kosloff [2], David P. Siderovski [1,*]

[1] Department of Pharmacology & Neuroscience, University of North Texas Health Science Center, Fort Worth, TX;
[2] Department of Human Biology, Faculty of Natural Sciences, University of Haifa, Israel;
[3] Atomwise Inc., San Francisco, CA;
[4] Department of Pathology, Carver College of Medicine, University of Iowa, Iowa City, IA;
[5] Department of Pharmaceutical Sciences, University of North Texas System College of Pharmacy, Fort Worth, TX.

* *Corresponding author*: UNTHSC, 3500 Camp Bowie Blvd, Fort Worth, TX 76107; david.siderovski@unthsc.edu

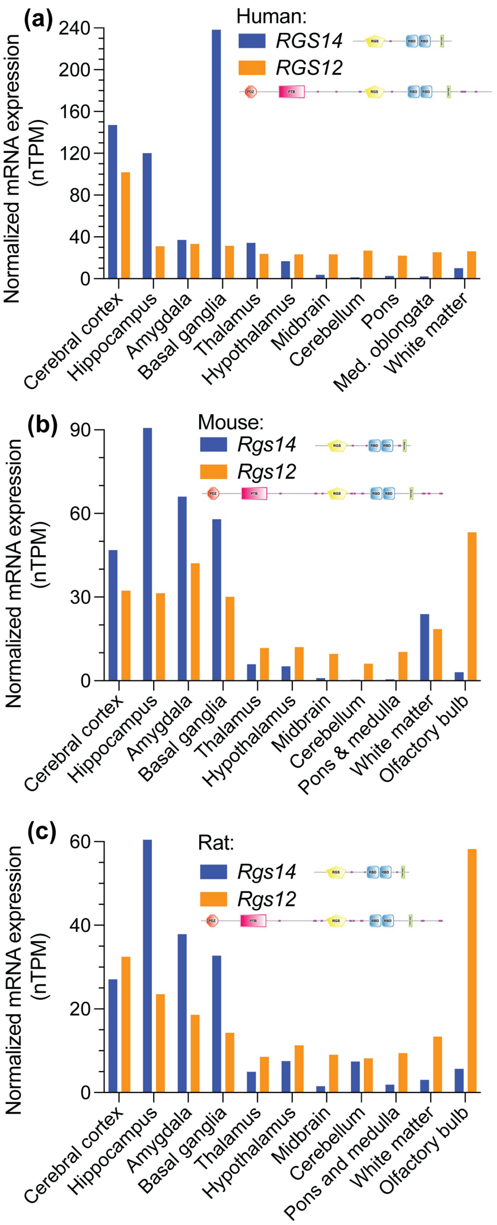

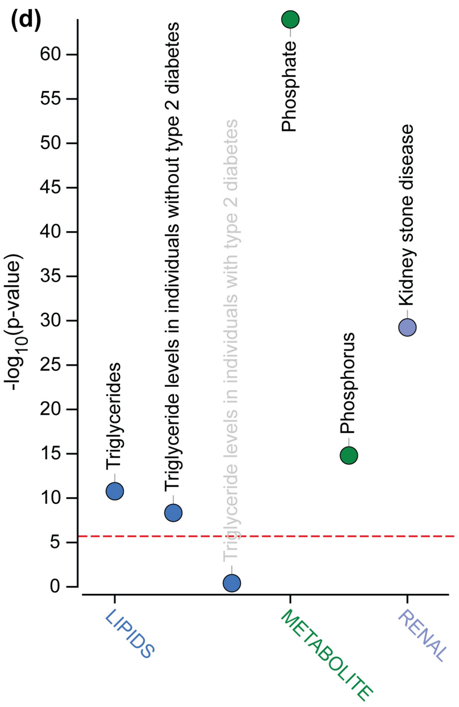

(e)

human gene	human subjects	sample ancestry	phenotype	p-value	zStat
RGS14	400,471	Mixed	Phosphate	1.4E-64	16.93
RGS14	70,082	Mixed	Kidney stone disease	7.2E-30	11.29
RGS14	42,793	Mixed	Phosphorus	2.0E-15	7.86
RGS14	2,011,284	Mixed	Triglycerides (TG)	2.1E-11	6.60
RGS14	402,944	Mixed	TG (non-T2D)	5.8E-09	5.71
RGS14	21,176	Mixed	TG (in T2D)	4.9E-01	0.02

Supplementary Figure S1. Comparative analyses of RGS14 and RGS12 protein domain organizations, brain-region gene expression patterns, and phenome-wide associations for human RGS14 genetic variation. Normalized transcript per million (nTPM) values are shown for RGS14 (blue bars) and RGS12 (orange bars) across homologous brain regions in (**a**) human, (**b**) mouse, and (**c**) rat. Human and mouse transcript expression data were obtained from the Human Protein Atlas (HPA) transcriptomics database (1) -- RGS14 (ENSG00000169220) and RGS12 (ENSG00000159788) -- each representing bulk RNA-seq expression across defined anatomical structures. Rat brain region data were curated from BioGPS BioDatabase System (2) dataset BDS_00054, which provides organ- and region-specific nTPM values derived from bulk RNA-seq (including discrete subregions such as the basolateral amygdala, corpus striatum, and subiculum). Region labels in the rat dataset were manually harmonized to match those in the human and mouse panels. Specifically, "corpus striatum" and "nucleus accumbens" were merged into the "Basal ganglia" category; "subiculum" was grouped with the "Hippocampus"; and "brainstem" was used to approximate "Pons and medulla". The olfactory bulb, absent from human HPA data, is shown for rat and mouse only. Expression values represent the arithmetic mean across multiple biological replicates per brain region, where applicable. Domain layouts within graph legends were generated from SMART outputs using the following UniProt entries: RGS14_HUMAN, RGS12_HUMAN, RGS14_MOUSE, RGS12_MOUSE, RGS14_RAT, and Swiss-Prot id O08774 for rat RGS12 protein sequence, respectively. Protein domains are color-coded: PDZ (orange hexagon), PTB (rose-pink rectangle), RGS-box (yellow pentagon), Ras-binding domain (RBD, cyan square), GoLoco (lime green rectangle), and predicted low-complexity regions (pink lines). Panels (**d**) and (**e**) represent graphical and numeric readouts, respectively, obtained from the Type 2 Diabetes Knowledge Portal (https://t2d.hugeamp.org; RRID: SCR_003743; ref. (3)) when queried for phenotypic associations to common human RGS14 gene sequence variations, calculated from bottom-line genetic associations using the MAGMA (Multi-marker Analysis of GenoMic Annotation) method (4). The generally accepted threshold for significance of MAGMA results ($p \leq 2.5 \times 10^{-6}$) is denoted with a red dotted line in the phenome-wide association (PheWAS) plot of panel (**d**), highlighting that the triglyceride levels in individuals without type 2 diabetes are significantly associated with human RGS14 gene variations (p-value 5.8×10^{-9}), but not triglyceride levels in individuals with type 2 diabetes. Sample size ("*human subjects*") in panel (**e**) refers to the number of individuals whose genotypes contributed to the bottom-line analysis of genetic associations for that phenotype. The MAGMA method's "Z-stat" is a standardized effect size from gene-level regression, indicating the strength and direction of association; positive values suggest stronger associations with increased trait values (4).

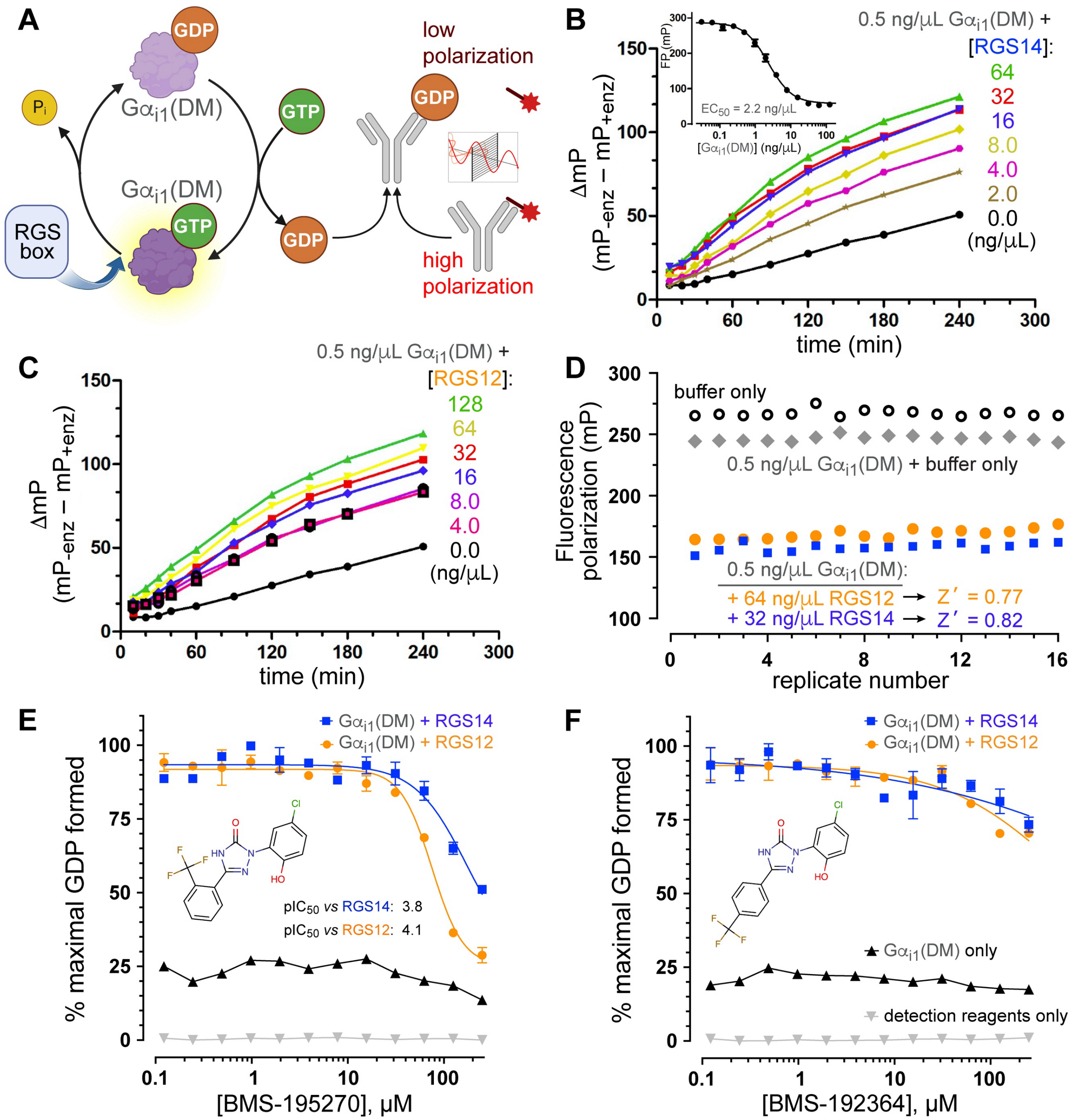

Supplementary Figure S2. Using the Transcreener® GDP RGScreen™ assay to measure $G\alpha_{i1}$(DM) GTPase activity in the presence of wild-type RGS14 and RGS12 RGS-box proteins. (A) Schematic representation of the Transcreener® GDP fluorescence polarization immunoassay as applied to measuring steady-state guanosine triphosphate (GTP) hydrolysis activity (and resultant GDP production) by a rate-altered, double point-mutant ("DM") form of $G\alpha_{i1}$ protein when its intrinsic GTP hydrolysis rate is accelerated by the GAP activity of an RGS-box protein (reaction details extensively explored in refs. (5, 6)). Fluorescent tracer is illustrated with a jagged red oval; when bound to the GDP-selective monoclonal antibody, emitted light remains polarized, whereas there is low polarization of emitted light when this tracer is displaced by free GDP. **(B, C)** Recombinant wild-type RGS14 RGS-box protein (panel B) and wild-type RGS12 RGS-box protein (panel C) are both observed, in a concentration-dependent fashion, to increase GDP production by 0.5 ng/μL $G\alpha_{i1}$(DM) protein (representing the EC_{20} for $G\alpha_{i1}$(DM) in a 20 μL final reaction volume), as measured by the change in polarization (ΔmP) at each indicated time point ("enz" = $G\alpha_{i1}$(DM) protein a.k.a. "enzyme"). *Inset to panel B:* Initial titration (to establish EC_{20}) of recombinant $G\alpha_{i1}$(DM) protein into Transcreener® GDP detection reagents and measurement of fluorescence polarization output (in units of milliP or "milli-polarization units") after reaction incubation for 120 minutes at 30 °C. **(D)** Sixteen (16) replicate measures of final fluorescence polarization were obtained after 180 min with indicated reaction conditions to calculate the assay's Z' factor: a dimensionless statistic (7) assessing both signal dynamic range and variation within data from both positive and negative controls. An observed Z' factor > 0.5 is generally considered permissible for assay launch in a screening campaign (7). **(E, F)** Tests of concentration-dependent inhibition of RGS14 (blue) and RGS12 (orange) RGS-box GAP activity on $G\alpha_{i1}$(DM) protein by Bristol Myers Squibb-identified 1,3-diaryl 1,2,4-(4H)-triazol-5-ones: BMS-195270 (panel E; estimated IC_{50}^{RGS14} of 174 μM [pIC_{50}^{RGS14} of 3.8] and estimated IC_{50}^{RGS12} of 75 μM [pIC_{50}^{RGS14} of 4.1] and BMS-192364 (panel F; considered inactive against RGS14 and RGS12)).

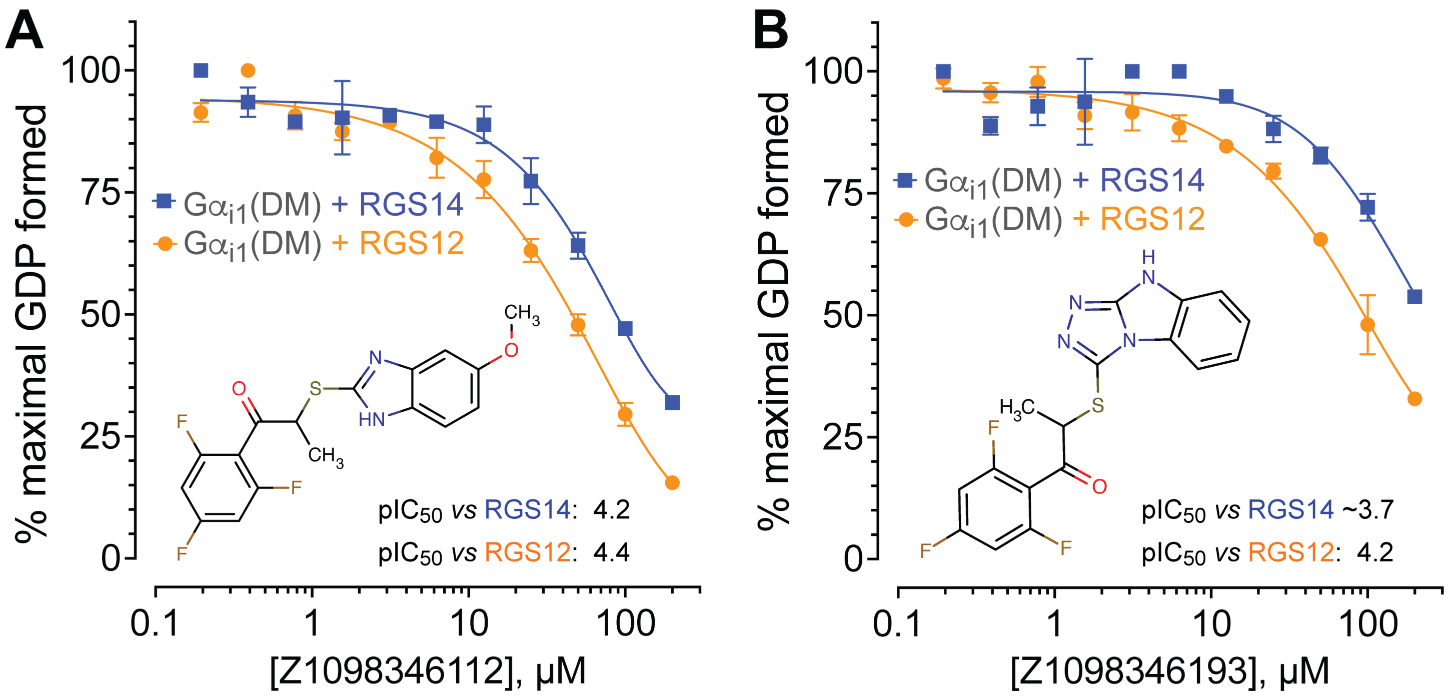

Supplementary Figure S3. RGS14 and RGS12 inhibition by initial hit compound Z1098346112 and the related compound Z1098346193. (**A**) Concentration-dependent inhibition of RGS14 (**blue**) and RGS12 (**orange**) RGS-box GAP activity by original hit compound 2-[(5-methoxy-1H-1,3-benzodiazol-2-yl)sulfanyl]-1-(2,4,6-trifluorophenyl) propane-1-one (Z1098346112; estimated pIC_{50}^{RGS14} of 4.2 and estimated IC_{50}^{RGS12} of 4.4) (from ref. (8)) and the sole Enamine-sourced analog found with activity, 2-(2,4,5,7-tetraaza-tricyclo[6.4.0.0^{2},^{6}]dodeca-1(12),3,5,8,10-pentaen-3-ylsulfanyl)-1-(2,4,6-trifluorophenyl)propan-1-one (Z1098346193; estimated pIC_{50}^{RGS14} of ~3.7 and estimated pIC_{50}^{RGS12} of 4.2), obtained after having screened >80 related, but ultimately inactive, benzimidazoles (listed in Supplementary Table S1).

SMILES	Enamine identifier	Tanimoto coefficient (ECFP4, *vs* Z1098346112)	pIC_{50} (RGS14)	2JNU pose 7 predicted Vina affinity (kcal/mol)
**Initial docking hit, initial followup active, and initial (negative) SAR around benzimidazoles**				
**COC1=CC2=C(C=C1)N=C(SC(C)C(=O)C1=C(F)C=C(F)C=C1F)N2**	**Z1098346112**	**1.00**	**4.2**	**-7.34**
**CC(SC1=NNC2=NC3=C(C=CC=C3)N21)C(=O)C1=C(F)C=C(F)C=C1F**	**Z1098346193**	**0.43**	**~3.7**	**-7.87**
COC1=CC=C(C(=O)C(C)SC2=NC3=C(C=C(OC)C=C3)N2)C=C1	Z16077705	0.71	<3.0	-6.88
COC1=CC2=C(C=C1)N=C(SC(C)C(=O)C1=CC=CC=C1)N2	Z16077228	0.69	<3.0	-6.92
COC1=CC2=C(C=C1)N=C(SCCC(=O)C1=CC=C(F)C=C1)N2	Z16077963	0.52	<3.0	-6.78
COC1=CC2=C(C=C1)N=C(SCC(=O)C1=CC=C(O)C=C1)N2	Z112468740	0.47	<3.0	-6.79
COC1=CC2=C(C=C1)N=C(SCC(O)C1=CC=CC=C1)N2	Z56784116	0.45	<3.0	-6.23
CCOC1=CC2=C(C=C1)NC(SCCC(=O)C1=CC=C(F)C=C1)=N2	Z19828508	0.33	<3.0	-6.51
**Benzimidazoles that interfere with Transcreener® GDP detection reagents**				
CC(SC1=NC=2C=CC=CC2N1C(F)F)C(=O)C=3C(F)=CC(F)=CC3F	Z1098351974	0.44	<3.0	-7.28
CCOC(=O)C=1C=CC=2N(CC)C(SC(C)C(=O)C=3C(F)=CC(F)=CC3F)=NC2C1	Z1098347818	0.42	<3.0	-7.02
CCCCN1C(SC(C)C(=O)C=2C(F)=CC(F)=CC2F)=NC=3C=C(C=CC13)S(=O)(=O)N(C)C	Z1098346992	0.36	<3.0	-7.27
CCOC(=O)C=1C=CC=2N(CC)C(SC(C)C(=O)C=3C=CC(NC(=O)C(C)C)=CC3)=NC2C1	Z65654925	0.24	<3.0	-6.94
**Benzimidazoles that exhibit non-specific inhibition (affecting Gαi1(DM) GTPase activity alone)**				
COC=1C=CC=2N=C(SC(C)C(=O)C=3C=CC(NC(=O)C)=CC3)NC2C1	Z16077185	0.62	<3.0	-7.20
COC=1C=CC=2N=C(SC(C)C(=O)C=3C=CC=4N(CCC4C3)C(=O)C)NC2C1	Z165591262	0.54	<3.0	-7.58
COC=1C=CC=2N=C(SCC(=O)C=3C=CC(F)=CC3)NC2C1	Z16077977	0.53	<3.0	-6.76
COC=1C=CC(=CC1)N2C(SC(C)C(=O)C=3C=C(C)C(C)=CC3C)=NC=4C=CC=CC24	Z19576531	0.32	<3.0	-7.23
CC(C)C=1C=CC(C(=O)C(C)SC2=NC=3C=CC=CC3N2C(C)C)=C(C1)C(C)C	Z54156331	0.26	<3.0	-7.43
CCC=1C=CC(=CC1)C(=O)C(C)SC2=NC=3C=C(C=CC3N2CC)S(=O)(=O)N	Z51418852	0.24	<3.0	-7.29
CCN1C(SC(C)C(=O)C=2C=CC=3N(CCC3C2)C(=O)C)=NC=4C=CC=CC14	Z165501154	0.23	<3.0	-7.04
**Inactive unsubstituted benzimidazoles**				
COC=1C=CC=2N=C(SC(C)C(=O)C=3C=CC(F)=CC3F)NC2C1	Z167736700	0.78	<3.0	-7.15
COC=1C=CC=2N=C(SC(C)C(=O)C=3C=CC(F)=CC3)NC2C1	Z16077798	0.75	<3.0	-7.05
COC=1C=CC=2N=C(SC(C)C(=O)C=3C=CC(F)=C(F)C3)NC2C1	Z16077536	0.73	<3.0	-7.41
COC=1C=CC=2N=C(SC(C)C(=O)C=3C=CC=C(F)C3)NC2C1	Z5032180758	0.73	<3.0	-7.03
COC=1C=CC=2N=C(SC(C)C(=O)NC=3C=CC(F)=CC3F)NC2C1	Z112468618	0.68	<3.0	-7.46
COC=1C=CC(C(=O)C(C)SC2=NC=3C=CC(OC)=CC3N2)=C(OC)C1	Z16077640	0.67	<3.0	-6.78
COC=1C=CC=2N=C(SC(C)C(=O)C=3C=CC(OC(F)F)=CC3)NC2C1	Z16077231	0.67	<3.0	-7.08
COC=1C=CC=2N=C(SC(C)C(=O)C=3C=C(C)C(C)=CC3C)NC2C1	Z16077531	0.66	<3.0	-6.91
COC=1C=CC=2N=C(SC(C)C(=O)C=3C=CC(Br)=CC3)NC2C1	Z16077584	0.66	<3.0	-6.86
CC(SC1=NC=2C=C(C)C=CC2N1)C(=O)C=3C(F)=CC(F)=CC3F	Z1098347474	0.65	<3.0	-7.41
COC=1C=CC=2N=C(SC(C)C(=O)NC=3C=C(F)C=CC3F)NC2C1	Z112468620	0.65	<3.0	-7.36
COC=1C=CC=2N=C(SC(C)C(=O)C=3C=CC(C)=C(C)C3)NC2C1	Z16077488	0.65	<3.0	-7.29
COC=1C=CC=2N=C(SC(C)C(=O)C=3C=CC(OC)=C(OC)C3)NC2C1	Z940567722	0.65	<3.0	-7.02
COC=1C=CC=2N=C(SC(C)C(=O)N(C)C)NC2C1	Z92077129	0.65	<3.0	-5.81
COC=1C=CC=2N=C(SC(C)C(=O)C=3C=CC=C(Cl)C3)NC2C1	Z16077229	0.64	<3.0	-6.98
COC=1C=CC=2N=C(SC(C)C(=O)C=3C=C(C)C=CC3C)NC2C1	Z16077483	0.64	<3.0	-6.98
CCC=1C=CC(=CC1)C(=O)C(C)SC2=NC=3C=CC(OC)=CC3N2	Z16077530	0.64	<3.0	-7.12
COC=1C=CC=2N=C(SC(C)C(=O)C=3C=CC(=CC3)C(C)(C)C)NC2C1	Z16077565	0.64	<3.0	-6.97
COC=1C=CC=2N=C(SC(C)C(=O)C3=CC=CO3)NC2C1	Z3277796272	0.64	<3.0	-6.69
COC=1C=CC=2N=C(SC(C)C(=O)C=3C=CC=CC3C(F)(F)F)NC2C1	Z5032180788	0.63	<3.0	-7.20
COC=1C=CC=2N=C(SC(C)C(=O)C=3NC(C)=C(C(=O)C)C3C)NC2C1	Z98717471	0.63	<3.0	-6.76
COC=1C=CC=2N=C(SC(C)C(=O)C=3C=CC=4CCCC4C3)NC2C1	Z16076970	0.61	<3.0	-7.53
COC=1C=CC=2N=C(SC(C)C(=O)C=3C=CC(NC(=O)C(C)C)=CC3)NC2C1	Z65841956	0.61	<3.0	-7.09
COC=1C=CC=2N=C(SC(C)C(=O)C=3C=CC(NC(=O)C(C)(C)C)=CC3)NC2C1	Z50965385	0.59	<3.0	-7.21
COC=1C=CC=2N=C(SC(C)C(=O)NC=3C=CC(C)=CC3)NC2C1	Z16077036	0.58	<3.0	-7.01
CCCC(=O)NC=1C=CC(=CC1)C(=O)C(C)SC2=NC=3C=CC(OC)=CC3N2	Z50965416	0.57	<3.0	-6.97
CCC(SC1=NC=2C=CC(OC)=CC2N1)C(=O)C=3C=CC(OC)=CC3	Z5032180767	0.56	<3.0	-6.87
COC=1C=CC=2N=C(SC(C(C)C)C(=O)OC(C)(C)C)NC2C1	Z5032180761	0.55	<3.0	-6.12
CCC(SC1=NC=2C=CC(OC)=CC2N1)C(=O)C=3C=CC=CC3	Z447938100	0.54	<3.0	-7.00
CCC(SC1=NC=2C=CC(OC)=CC2N1)C(=O)C=3C=CC(Cl)=CC3	Z5032180785	0.52	<3.0	-6.87
CC(SC1=NC=2C=C(C)C=CC2N1)C(=O)C=3C=CC(F)=CC3F	Z167804418	0.50	<3.0	-7.24
CCC(SC1=NC=2C=CC(OC)=CC2N1)C(=O)C=3C=CC(NC(=O)C)=CC3	Z1340653159	0.49	<3.0	-7.33
COC=1C=CC=2NC(SC(C(=O)N)C=3C=CC(F)=CC3)=NC2C1	Z234729962	0.49	<3.0	-7.31
CC(SC1=NC=2C=C(C)C=CC2N1)C(=O)C=3C=CC(F)=CC3	Z18443583	0.48	<3.0	-7.17
CC(SC1=NC=2C=C(C)C=CC2N1)C(=O)C=3C=CC(OC(F)F)=CC3	Z18443016	0.46	<3.0	-7.19
CC(SC1=NC=2C=C(C)C=CC2N1)C(=O)C=3C=CC=C(F)C3	Z5032180743	0.46	<3.0	-7.16
COC=1C=CC(=CC1Cl)C(=O)C(C)SC2=NC=3C=C(C)C=CC3N2	Z5032180749	0.43	<3.0	-7.27
CCC=1C=CC(=CC1)C(=O)C(C)SC2=NC=3C=C(C)C=CC3N2	Z18443315	0.39	<3.0	-7.27
COC=1C=CC=2N=C(SCCOCC3CCCO3)NC2C1	Z1258859165	0.38	<3.0	-5.82
COC=1C=CC=2NC(SCC(=O)C=3C=CC(O)=C(O)C3)=NC2C1	Z223822242	0.37	<3.0	-7.02
CCOC=1C=CC=2N=C(SCC(=O)C=3C=CC(CC)=CC3)NC2C1	Z55030990	0.36	<3.0	-6.71
CC(SC1=NC=2C=C(C)C=CC2N1)C(=O)C=3C=CC=4N(CCC4C3)C(=O)C	Z165332254	0.34	<3.0	-7.68
**Inactive N-substituted benzimidazoles**				
COC=1C=CC(=CC1)N2C(SC(C)C(=O)C=3C(F)=CC(F)=CC3F)=NC=4C=CC=CC24	Z5032180751	0.52	<3.0	-7.27
CCN1C(SC(C)C(=O)C=2C(F)=CC(F)=CC2F)=NC=3C=CC=CC13	Z1098345881	0.45	<3.0	-6.94
CCN1C(SC(C)C(=O)C=2C(F)=CC(F)=CC2F)=NC=3C=C(C=CC13)S(=O)(=O)N4CCCCC4	Z5032180738	0.38	<3.0	-7.73
CCN1C(SC(C)C(=O)C=2C=CC(OC)=CC2)=NC=3C=C(C=CC13)S(=O)(=O)N	Z51419018	0.33	<3.0	-7.01
COC=1C=CC(=CC1)N2C(SC(C)C(=O)C=3C=C(C)C=CC3C)=NC=4C=CC=CC24	Z19576483	0.32	<3.0	-6.73
COCCN1C(SC(C)C(=O)C=2C=CC(OC)=CC2)=NC=3C=CC=CC13	Z16216221	0.32	<3.0	-6.59
CCN1C(SC(C)C(=O)C=2C=CC(F)=CC2)=NC=3C=C(C=CC13)S(=O)(=O)N	Z51419107	0.29	<3.0	-7.05
COC=1C=CC(=CC1)C(SC2=NC=3C=CC=CC3N2C)C(=O)C=4C=CC(OC)=CC4	Z15478257	0.28	<3.0	-7.43
CCOC=1C=CC(=CC1)C(=O)C(C)SC2=NC=3C=C(C=CC3N2CC)S(=O)(=O)N	Z51419689	0.28	<3.0	-6.80
CCOC=1C=CC(=CC1)C(=O)C(C)SC2=NC=3C=CC=CC3N2C	Z15479255	0.27	<3.0	-6.77
COCCN1C(SC(C)C(=O)C=2C=CC(Br)=CC2)=NC=3C=CC=CC13	Z16216100	0.25	<3.0	-6.51
CCCCN1C(SC(C(=O)C=2C=CC(OC)=CC2)C=3C=CC(OC)=CC3)=NC=4C=C(C=CC14)S(=O)(=O)N	Z19162246	0.24	<3.0	-7.01
CCN1C(SC(C)C(=O)C=2C=CC(Cl)=CC2)=NC=3C=C(C=CC13)S(=O)(=O)N	Z51418567	0.24	<3.0	-7.06
CCN1C(SC(C)C(=O)C=2C=CC(Br)=CC2)=NC=3C=C(C=CC13)S(=O)(=O)N	Z51418902	0.24	<3.0	-7.04
CCN1C(SC(C)C(=O)C=2C=CC=C(Cl)C2)=NC=3C=C(C=CC13)S(=O)(=O)N	Z51418566	0.23	<3.0	-6.98
CCN1C(SC(C)C(=O)C=2C=C(C)C=CC2C)=NC=3C=C(C=CC13)S(=O)(=O)N	Z51418807	0.23	<3.0	-6.54
CCN1C(SC(C)C(=O)C=2C=CC(NS(=O)(=O)C)=CC2)=NC=3C=CC=CC13	Z50932618	0.23	<3.0	-6.73
COCCN1C(SC(C)C(=O)C=2C=CC=3N(CCC3C2)C(=O)C)=NC=4C=CC=CC14	Z165550978	0.23	<3.0	-7.00
CCN1C(SC(C)C(=O)C=2C=CC(NC(=O)C)=CC2)=NC=3C=C(C=CC13)S(=O)(=O)N	Z51418527	0.23	<3.0	-7.23
CCN1C(SC(C)C(=O)C=2C=CC=3CCCC3C2)=NC=4C=C(C=CC14)S(=O)(=O)N	Z51418316	0.22	<3.0	-7.13
CCN1C(SC(C)C(=O)C=2C=CC=CC2)=NC=3C=C(C=CC13)S(=O)(=O)N4CCOCC4	Z16669878	0.22	<3.0	-7.43
CC(SC1=NC=2C=CC=CC2N1C)C(=O)C=3C=CC(NS(=O)(=O)C)=CC3	Z50932699	0.22	<3.0	-6.93
CCN1C(SC(C)C(=O)C=2C=CC=3CCCC3C2)=NC=4C=C(C=CC14)S(=O)(=O)N5CCOCC5	Z16669629	0.21	<3.0	-7.57

Supplementary Table S1. Chemical, computational, and experimental characteristics of benzimidazole-based RGS14 inhibitor candidates. This table summarizes the structural and screening data for benzimidazole derivatives tested during early structure-activity relationship (SAR) exploration. Columns include: the compound SMILES string, the Enamine identifier, the Tanimoto similarity coefficient (ECFP4) *versus* initial active benzimidazole derivative Z1098346112, experimentally determined pIC_{50} values against RGS14-catalyzed GTP hydrolysis (where available), and the AutoDock Vina-predicted binding affinity (in kcal/mol) for pose 7 of the RGS14 RGS-box (PDB id 2JNU). Compounds are grouped by classification based on experimental outcomes, including initial actives, signal-interfering compounds, non-specific inhibitors of $G\alpha_{i1}$(DM) GTPase activity, and inactive analogs with either unsubstituted or N-substituted benzimidazole scaffolds. Where applicable, lack of pIC_{50} values reflects either inactivity or interference with assay components.

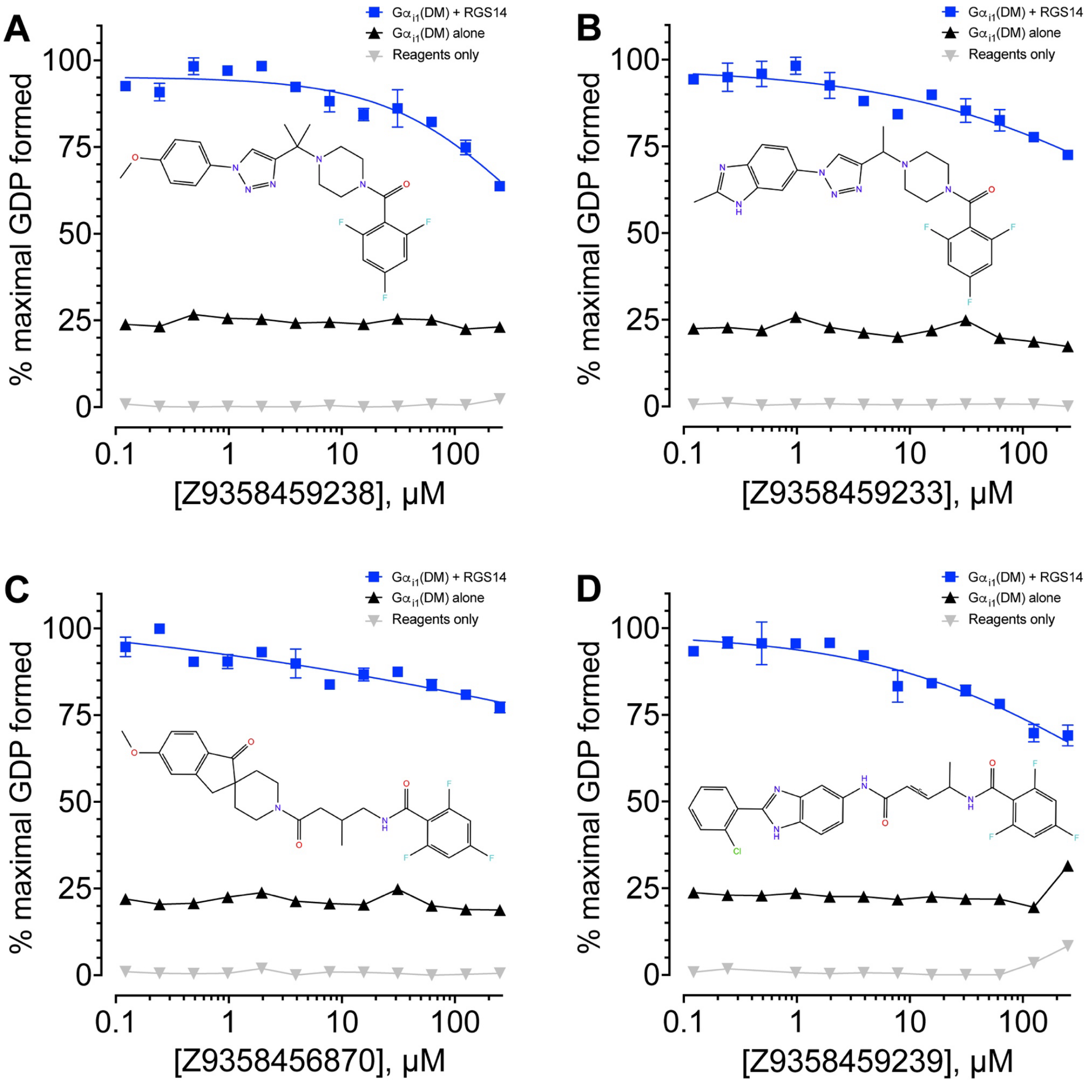

Supplementary Figure S4. Multi-ringed 2,4,6-trifluorobenzoyl analogs fail to inhibit RGS14 GAP activity.
(**A-D**) Transcreener® GDP-detection RGScreen™ assays assessing the effect of four multi-ringed, non-benzimidazole analogs -- each containing a 2,4,6-trifluorobenzoyl substituent -- on GTP hydrolysis catalyzed by $G\alpha_{i1}$(DM) protein in the presence or absence of recombinant RGS14 RGS-box protein. Structures were selected based on shared substructure with the active benzimidazole compounds Z1098346112 and Z1098346193, both of which possess a (2,4,6-trifluorophenyl)propan-1-one moiety. Despite their similar size and functional group presentation, none of these four analogs exhibited meaningful inhibition of RGS14-accelerated GAP activity across the tested concentration range. Negative controls ($G\alpha_{i1}$(DM) alone and reagents only) are shown for comparison.

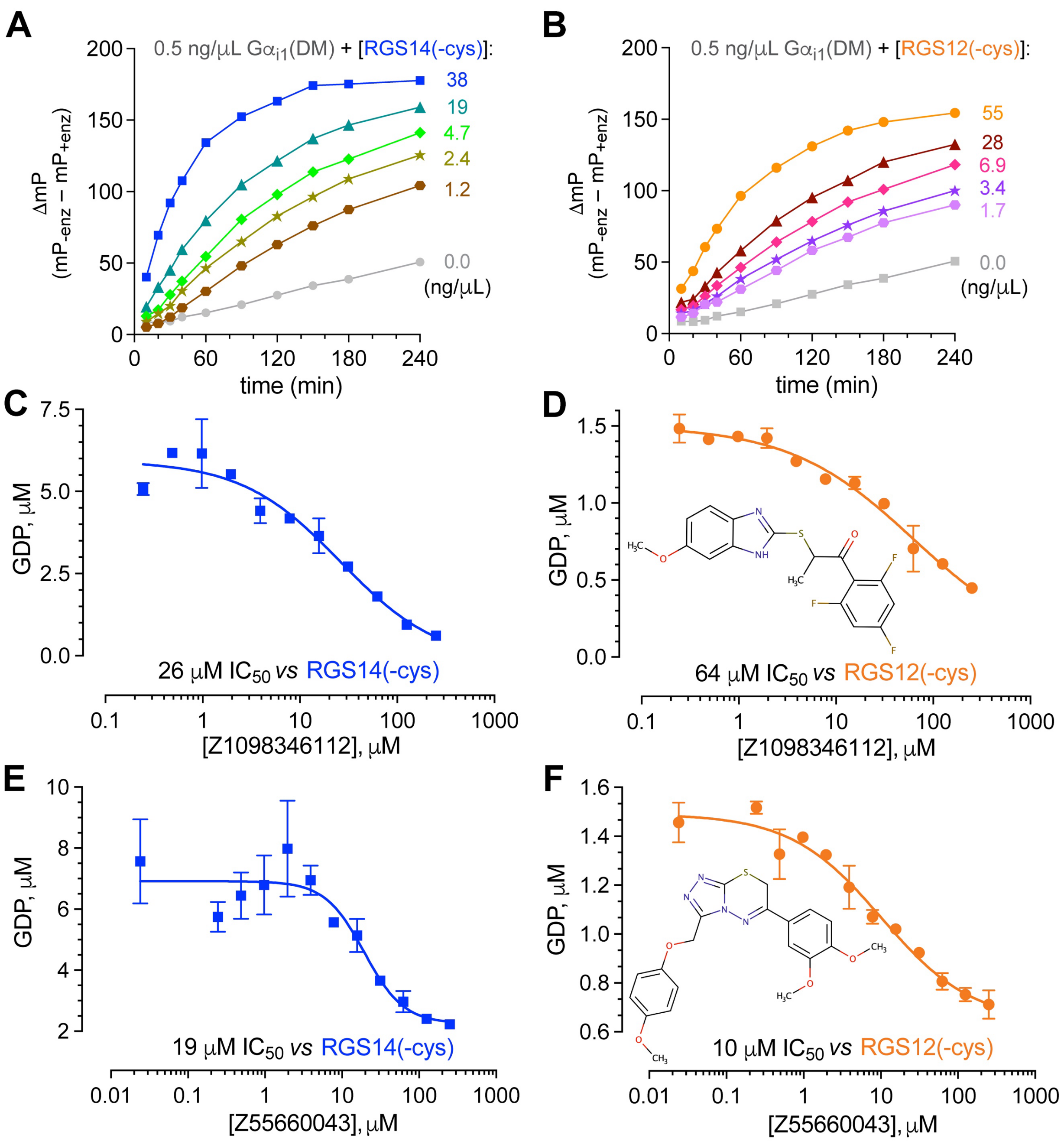

Supplementary Figure S5. RGS-box inhibitors of the benzimidazole and 1,2,4-triazolo[3,4-b][1,3,4]thiadiazine series inhibit the GAP activities of cysteine-less RGS-box proteins. (A,B) Recombinant, cysteine residue-lacking ("-cys") RGS14 RGS-box protein (panel A) and cysteine residue-lacking RGS12 RGS-box protein (panel A) were both observed, in a concentration-dependent fashion (indicated in ng/μL by color), to increase steady-state GDP production by 0.5 ng/μL $G\alpha_{i1}$(DM) protein in a 20 μL final reaction volume, as measured by the Transcreener® GDP-detection RGScreen™ assay and detected by the change in polarization (ΔmP) at each indicated time point ("enz" = $G\alpha_{i1}$(DM) protein a.k.a. "enzyme"). (**C,D**) End-point GDP production measured at 120 min in the presence of indicated concentrations of initial hit compound Z1098346112, as produced by the $G\alpha_{i1}$(DM) subunit and accelerated by the GAP activity of cysteine residue-lacking RGS14 RGS-box protein ("RGS14(-cys)"; panel C) or an RGS12 RGS-box recombinant protein lacking all wild-type cysteine residues ("RGS12(-cys)"; panel D). (**E,F**) GDP production in the presence of indicated concentrations of triazolothiadiazine compound Z55660043, as produced in reactions similar to panels C and D, respectively. Using standard curves of mP signal from defined GDP input, Transcreener® GDP detection output was converted to GDP produced (y-axes).

		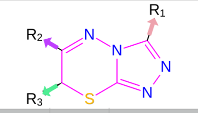														
SMILES	Enamine Name	R3	R2	R1	RGS14 pIC50	RGS12 pIC50	Ratio	MW (Da)	cLogP (o/w)	TPSA (Å²)	accept H-bond	donor H-bond	Rotatable bond count	Fsp³		
O=C([O-])C[C@@H]1Sc2nnc(COc3ccc(Cl)cc3)n2N=C1c1ccccc1	Z90276197	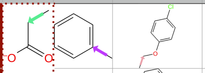			4.09	4.37	0.94	414.9	2.171	89.3	6	2	6	0.11		
O=C([O-])C[C@@H]1Sc2nnc([C@H]3COc4ccccc4O3)n2N=C1c1cccs1	Z90275816	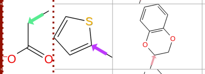			4.69	4.35	**1.08**	414.5	1.362	98.5	7	2	4	0.17		
COc1ccc(Cc2nnc3n2N=C(c2ccccc2)[C@H](CC(=O)[O-])S3)cc1OC	Z90277093	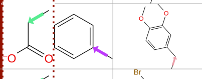			4.56	5.17	0.88	424.5	1.569	98.5	7	2	4	0.19		
O=C([O-])C[C@@H]1Sc2nnc(Cc3ccc(Br)cc3)n2N=C1c1ccc(Cl)cc1	Z90276771	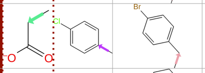			4.53	4.96	0.91	477.8	3.108	80.0	5	2	5	0.11		
O=C([O-])C[C@@H]1Sc2nnc(CCC3CCCCC3)n2N=C1c1ccccc1	Z90277285	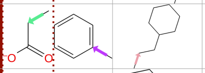			4.42	4.62	0.96	384.5	2.796	80.0	5	2	6	0.45		
COc1ccccc1-c1nnc2n1N=C(c1cccs1)[C@@H](CC(=O)[O-])S2	Z90275304	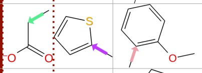			4.28	4.42	0.97	386.4	1.707	89.3	6	2	5	0.12		
O=C([O-])C[C@@H]1Sc2nnc([C@@H]3CCCO3)n2N=C1c1ccc(Cl)cc1	Z90278051	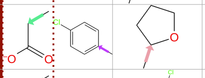			4.25	3.97	**1.07**	378.8	1.097	89.3	6	2	4	0.31		
Cc1ccc(C2=Nn3c(COc4ccc(Cl)cc4)nnc3S[C@@H]2CC(=O)[O-])cc1	Z90276194	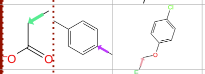			4.21	4.21	**1.00**	428.9	2.684	89.3	6	2	6	0.15		
Cc1ccc(C2=Nn3c(Cc4ccc(F)cc4)nnc3S[C@@H]2CC(=O)[O-])cc1	Z90278242	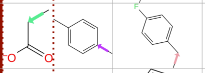			4.11	4.19	0.98	396.4	2.540	80.0	5	2	5	0.15		
O=C([O-])C[C@@H]1Sc2nnc(-c3ccco3)n2N=C1c1ccccc1	Z90276389	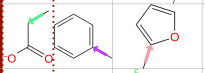			4.10	4.08	**1.00**	340.4	1.012	93.2	5	2	4	0.06		
O=C([O-])C[C@H]1Sc2nnc(Cc3ccc(F)cc3)n2N=C1c1cccs1	Z90278248	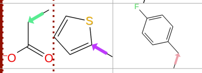			4.09	4.27	0.96	388.4	1.940	80.0	5	2	5	0.12		
O=C([O-])C[C@@H]1Sc2nnc(C3CC3)n2N=C1c1ccc(F)cc1	Z90274599	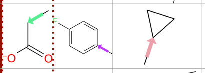			4.04	4.38	0.92	332.4	0.972	80.0	5	2	4	0.27		
CCN(CC)S(=O)(=O)c1cccc(-c2nnc3n2N=C(c2ccc(C)cc2)[C@@H](CC(=O)[O-])S3)c1	Z90274338	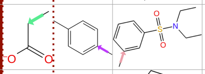			4.03	3.54	**1.14**	499.6	2.067	117.4	7	2	7	0.26		
O=C([O-])C[C@@H]1Sc2nnc(-c3ccco3)n2N=C1c1ccc(F)cc1	Z90276391	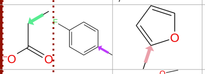			4.03	4.21	0.96	358.3	1.154	93.2	5	2	4	0.06		
COc1ccc(-c2nnc3n2N=C(c2cccs2)[C@H](CC(=O)[O-])S3)cc1	Z90275560	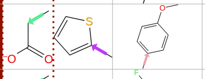			3.98	4.32	0.92	386.4	1.707	89.3	6	2	5	0.12		
O=C([O-])C[C@H]1Sc2nnc(Cc3ccc(F)cc3)n2N=C1c1ccccc1	Z90278245	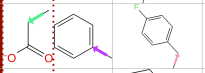			3.97	4.24	0.94	382.4	2.027	80.0	5	2	5	0.11		
COc1ccccc1-c1nnc2n1N=C(c1ccc(Cl)cc1)[C@H](CC(=O)[O-])S2	Z90275299	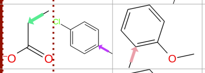			3.91	4.14	0.94	414.9	2.160	89.3	6	2	5	0.11		
COc1ccccc1-c1nnc2n1N=C(c1ccccc1)[C@H](CC(=O)[O-])S2	Z90275301	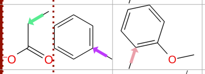			3.81	4.31	0.88	380.4	1.633	89.3	6	2	5	0.11		
COc1cccc(OCc2nnc3n2N=C(c2ccccc2)[C@H](CC(=O)[O-])S3)c1	Z90278437	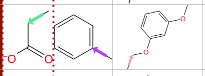			3.77	3.76	**1.00**	410.4	1.409	98.5	7	2	7	0.15		
Cc1ccc(C2=Nn3c(nnc3C3CC3)S[C@H]2CC(=O)[O-])cc1	Z90274594	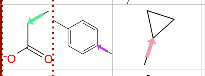			3.56	4.18	0.85	328.4	1.343	80.0	5	2	4	0.31		
O=C([O-])C[C@@H]1Sc2nnc(C3CC3)n2N=C1c1ccccc1	Z90274597	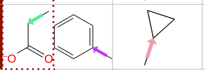			3.52	3.82	0.92	314.4	0.829	80.0	5	2	4	0.27		

Supplementary Figure S6. Activity and computed physicochemical properties of triazolothiadiazine derivatives bearing a carboxylic acid R3-group. This panel displays a collection of active triazolothiadiazine compounds that share a carboxylic acid functional group at the R3 position. Each compound is shown with its SMILES string and annotated with its Enamine identifier, R-group substitutions, and experimentally measured RGS14 and RGS12 pIC_{50} values (absolute $\log_{10}$ of the compound concentration yielding 50% inhibition in the Transcreener® GDP-detection RGScreen™ assay). The selectivity ratio (RGS14 pIC_{50} / RGS12 pIC_{50}) is included, with values >1.0 (bold) indicating preferential inhibition of RGS14. Additional physicochemical descriptors were computed using RDKit Chem Descriptors: molecular weight (MW, in Daltons), predicted octanol/water partition coefficient (cLogP), total polar surface area (TPSA, Å²), number of hydrogen bond acceptors (HBA) and donors (HBD), rotatable bond count, and the fraction of sp^3-hybridized carbon atoms (Fsp^3). This dataset highlights both the structural diversity and selective activity retained within the subset of acidic analogs.

SMILES	Enamine Name	R3	R2 / R1 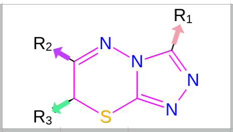	RGS14 pIC50	RGS12 pIC50	Ratio	MW (Da)	cLogP (o/w)	TPSA (Å²)	accept H-bond	donor H-bond	Rotatable bond count	Fsp³
COc1ccc(OCc2nnc3n2N=C(c2ccc(OC)c(OC)c2)CS3)cc1	Z55660043	H	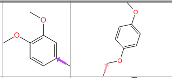	**5.64**	5.92	0.95	412.5	1.586	79.7	7	1	7	0.20
Cc1ccc(C2=Nn3c(COc4ccccc4)nnc3SC2)c(C)c1	Z55659515	H	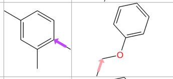	5.39	5.39	**1.00**	350.4	3.086	52.0	4	1	4	0.16
c1ccc2c(c1)OC[C@@H](c1nnc3n1N=C(c1cc4ccccc4o1)CS3)O2	Z55628018	H	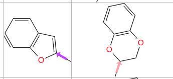	4.84	5.24	0.92	390.4	2.021	74.3	5	1	2	0.10
CCCc1ccc(C2=Nn3c(COc4ccccc4F)nnc3SC2)cc1	Z55660462	H	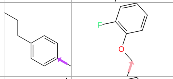	4.78	4.98	0.96	382.5	3.605	52.0	4	1	6	0.20
COc1ccccc1C1=Nn2c(COc3ccccc3)nnc2SC1	Z56944652	H	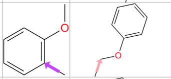	4.72	5.47	0.86	352.4	1.902	61.2	5	1	5	0.11
c1ccc2c(c1)OC[C@@H](c1nnc3n1N=C(c1ccc4c(c1)OCCO4)CS3)O2	Z55660304	H	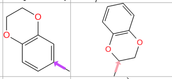	4.54	5.06	0.90	408.4	1.455	79.7	7	1	2	0.20
COc1ccc(OCc2nnc3n2N=C(c2cc4ccccc4o2)CS3)cc1	Z55627844	H	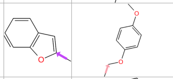	5.44	4.66	1.17	392.4	1.981	74.3	5	1	5	0.10
FC(F)Oc1ccc(C2=Nn3c(COc4ccccc4)nnc3SC2)cc1	Z56840012	H	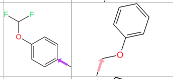	4.44	4.94	0.90	388.4	2.828	61.2	5	1	6	0.11
CC(C)(C)c1ccc(C2=Nn3c(nnc3-c3ccco3)SC2)cc1	Z55660110	H	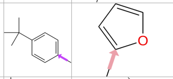	4.25	4.27	**1.00**	338.4	3.049	55.9	3	1	3	0.22
COc1ccc(OCc2nnc3n2N=C(c2ccc(OC)cc2OC)CS3)cc1	Z103673844	H	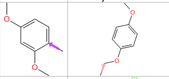	4.09	4.35	0.94	412.5	1.586	79.7	7	1	7	0.20
Cc1ccc(C2=Nn3c(COc4ccc(Cl)cc4)nnc3SC2)c(C)c1	Z55660524	H	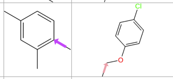	3.77	4.57	0.82	384.9	3.690	52.0	4	1	4	0.16
CC(C)c1nnc2n1N=C(c1cc3ccccc3o1)CS2	Z55627782	H	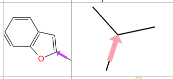	3.72	4.56	0.82	298.4	1.865	55.9	3	1	2	0.20
COc1cccc(C2=Nn3c(COc4ccc(Cl)cc4)nnc3SC2)c1	Z55660496	H	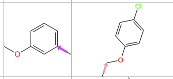	3.72	4.37	0.85	386.9	2.506	61.2	5	1	5	0.11
COc1ccc(OCc2nnc3n2N=C(c2cc(C)n(-c4nccs4)c2C)CS3)cc1	Z667824954	H	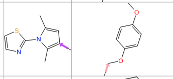	3.66	4.19	0.87	452.5	2.605	79.0	6	1	6	0.19
Fc1ccccc1OCc1nnc2n1N=C(c1ccc(Cl)cc1)CS2	Z55660438	H	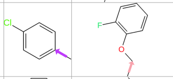	3.63	4.69	0.77	374.8	2.806	52.0	4	1	4	0.06
COc1ccc(OCc2nnc3n2N=C(c2cccc(N4CCCC4=O)c2)CS3)cc1	Z667824974	H	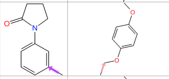	3.19	3.65	0.87	435.5	1.230	81.5	6	1	6	0.23
COc1ccc(OCc2nnc3n2N=C(c2ccc(NC(C)=O)cc2)CS3)cc1	Z55660046	H	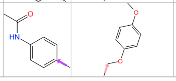	< 3.00	3.81		409.5	1.139	90.3	6	2	6	0.15

Supplementary Figure S7. Activity and computed physicochemical properties of triazolothiadiazine derivatives lacking an R3-group. This panel displays a collection of active triazolothiadiazine compounds that lack a functional group at the R3 position. Each compound is shown with its SMILES string and annotated with its Enamine identifier, R-group substitutions, and experimentally measured RGS14 and RGS12 pIC_{50} values (absolute log_{10} of the compound concentration yielding 50% inhibition in the Transcreener® GDP-detection RGScreen™ assay). The selectivity ratio (RGS14 pIC_{50} / RGS12 pIC_{50}) is included, with values equal to 1.0 (bold) indicating no preferential inhibition of RGS14 or RGS12. Additional physicochemical descriptors were computed using RDKit Chem Descriptors: molecular weight (MW, in Daltons), predicted octanol/water partition coefficient (cLogP), total polar surface area (TPSA, Å²), number of hydrogen bond acceptors (HBA) and donors (HBD), rotatable bond count, and the fraction of sp^3-hybridized carbon atoms (Fsp^3).

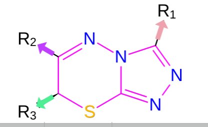

SMILES	Enamine Name	R3	R2	R1	RGS14 pIC50	RGS12 pIC50	Ratio	MW (Da)	cLogP (o/w)	TPSA (Å²)	accept H-bond	donor H-bond	Rotatable bond count	Fsp³
COC(=O)[C@@H]1Sc2nnc(COc3ccc(OC)cc3)n2N=C1c1cccc(N2CCCC2=O)c1	Z7976707565	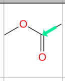	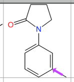	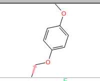	4.56	4.00	**1.14**	493.5	1.863	108.1	7	0	8	0.29
COC(=O)[C@H]1Sc2nnc(COc3ccc(F)cc3)n2N=C1c1cc2ccccc2o1	Z8201263931	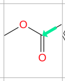	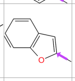	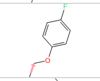	4.00	4.39	0.91	438.4	2.235	91.4	5	1	6	0.10
COc1ccc(OCc2nnc3n2N=C(c2ccc(C)cc2)[C@@H](C)S3)cc1	Z55660052	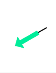	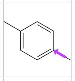	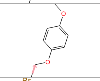	4.69	5.44	0.86	380.5	2.564	61.2	5	1	5	0.20
C[C@H]1Sc2nnc(Cc3ccc(Br)cc3)n2N=C1c1ccc(Cl)cc1	Z55660592	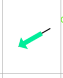	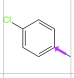	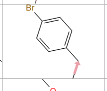	4.67	5.00	0.93	433.8	3.899	42.7	3	1	3	0.11
COc1ccc(-c2nnc3n2N=C(c2ccc(NC(C)=O)cc2)[C@@H](C)S3)cc1OC	Z90277200	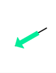	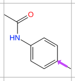	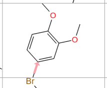	4.52	4.73	0.96	423.5	1.516	90.3	6	2	5	0.19
C[C@H]1Sc2nnc(Cc3ccc(Br)cc3)n2N=C1c1ccc([N-]S(C)(=O)=O)cc1	Z90276813	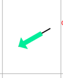	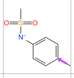	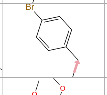	4.14	4.87	0.85	492.4	1.668	88.9	5	2	4	0.16
COc1ccc(Cc2nnc3n2N=C(c2ccc(C)c(C)c2)[C@@H](C)S3)cc1OC	Z55660805	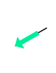	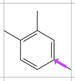	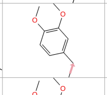	4.12	4.53	0.91	408.5	3.237	61.2	5	1	5	0.27
COc1ccc(Cc2nnc3n2N=C(c2ccccc2)[C@@H](C)S3)cc1OC	Z55660788	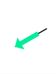	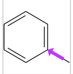	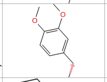	4.03	4.50	0.90	380.5	2.210	61.2	5	1	5	0.20
CCOc1ccccc1-c1nnc2n1N=C(c1ccc(NC(=O)C(C)C)cc1)[C@@H](C)S2	Z90278674	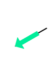	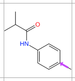	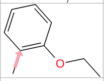	3.99	4.35	0.92	435.5	3.274	81.1	5	2	6	0.26
CSc1nnc2n1N=C(c1ccc(NC(=O)C(C)C)cc1)[C@H](C)S2	Z90277842	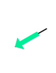	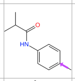	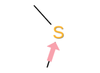	3.97	4.21	0.94	361.5	3.275	72.2	4	1	4	0.37
C1CC[C@H]2Sc3nnc([C@H]4CCCO4)n3N=C2C1	Z90278035	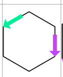	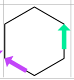	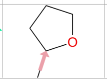	4.21	4.52	0.93	264.3	1.382	52.3	4	0	1	0.75

Supplementary Figure S8. Activity and computed physicochemical properties of triazolothiadiazine derivatives with R3-groups different than a carboxylic-acid moiety. This panel displays a collection of active triazolothiadiazine compounds that possess various different functional groups at the R3 position. Each compound is shown with its SMILES string and annotated with its Enamine identifier, R-group substitutions, and experimentally measured RGS14 and RGS12 pIC_{50} values (absolute log_{10} of the compound concentration yielding 50% inhibition in the Transcreener® GDP-detection RGScreen™ assay). The selectivity ratio (RGS14 pIC_{50} / RGS12 pIC_{50}) is included, with values >1.0 (bold) indicating preferential inhibition of RGS14. Additional physicochemical descriptors were computed using RDKit Chem Descriptors: molecular weight (MW, in Daltons), predicted octanol/water partition coefficient (cLogP), total polar surface area (TPSA, Å²), number of hydrogen bond acceptors (HBA) and donors (HBD), rotatable bond count, and the fraction of sp³-hybridized carbon atoms (Fsp³).

Supplementary Table S2. List of 34 triazolothiadiazines tested for RGS-box inhibitory activity and found inactive *in vitro*.

SMILES	Name	Vina affinity (kcal/mol)*	logS @ pH 7.4§	IUPAC name
Cc1ccc(C2=Nn3c(nnc3[C@@H]3COc4ccccc4O3)S[C@H]2C)cc1C	Z55660317	-8.81	-7.36	(7S)-3-[(2R)-2,3-dihydro-1,4-benzodioxin-2-yl]-6-(3,4-dimethylphenyl)-7-methyl-7H-[1,2,4]triazolo[3,4-b][1,3,4]thiadiazine
COc1cccc(OCc2nnc3n2N=C(c2ccccc2)[C@@H](C(=O)Nc2ccc4c(c2)OCO4)S3)c1	Z90278454	-8.27	-6.65	N-(2H-1,3-benzodioxol-5-yl)-3-[(3-methoxyphenoxy)methyl]-6-phenyl-5H-[1,2,4]triazolo[3,4-b][1,3,4]thiadiazine-7-carboxamide
CCc1cccc2c(C3=Nn4c(COc5ccc(OC)cc5)nnc4S[C@H]3c3ccccc3)c[nH]c12	Z90276677	-8.23	-7.67	7-ethyl-3-{3-[(4-methoxyphenoxy)methyl]-7-phenyl-5H-[1,2,4]triazolo[3,4-b][1,3,4]thiadiazin-6-yl}-1H-indole
Cc1ccc(C2=Nn3c(nnc3[C@H]3COc4ccccc4O3)S[C@H]2CC(=O)[O-])cc1	Z90275810	-8.20	-1.51	2-{3-[(2S)-2,3-dihydro-1,4-benzodioxin-2-yl]-6-(4-methylphenyl)-5H-[1,2,4]triazolo[3,4-b][1,3,4]thiadiazin-7-yl}acetic acid
Fc1ccc(OCc2nnc3n2N=C(c2cc4ccccc4o2)CS3)cc1	Z141354104	-8.13	-6.12	6-(1-benzofuran-2-yl)-3-[(4-fluorophenoxy)methyl]-5H-[1,2,4]triazolo[3,4-b][1,3,4]thiadiazine
COc1ccc(OCc2nnc3n2N=C(c2ccc4c(c2)NC(=O)CO4)CS3)cc1	Z55627846	-8.12	-4.86	6-{3-[(4-methoxyphenoxy)methyl]-5H-[1,2,4]triazolo[3,4-b][1,3,4]thiadiazin-6-yl}-3,4-dihydro-2H-1,4-benzoxazin-3-one
Cc1ccc(C2=Nn3c(COc4c(Cl)cccc4Cl)nnc3S[C@@H]2C)cc1C	Z141361328	-8.03	-8.24	(7R)-3-[(2,6-dichlorophenoxy)methyl]-6-(3,4-dimethylphenyl)-7-methyl-7H-[1,2,4]triazolo[3,4-b][1,3,4]thiadiazine
c1ccc(C2=Nn3c(Cc4cccc5ccccc45)nnc3SC2)cc1	Z55659738	-7.97	-6.38	3-[(naphthalen-1-yl)methyl]-6-phenyl-5H-[1,2,4]triazolo[3,4-b][1,3,4]thiadiazine
COc1ccc(C2=Nn3c(nnc3[C@@H]3COc4ccccc4O3)SC2)cc1OC	Z55660314	-7.95	-5.21	3-[(2R)-2,3-dihydro-1,4-benzodioxin-2-yl]-6-(3,4-dimethoxyphenyl)-5H-[1,2,4]triazolo[3,4-b][1,3,4]thiadiazine
COc1ccc(Cc2nnc3n2N=C(c2ccc(Cl)cc2)[C@@H](C)S3)cc1OC	Z55660789	-7.93	-5.13	6-(4-chlorophenyl)-3-[(3,4-dimethoxyphenyl)methyl]-7-methyl-5H-[1,2,4]triazolo[3,4-b][1,3,4]thiadiazine
COc1cccc(OCc2nnc3n2N=C(c2c(C)[nH]c4ccccc24)[C@H](C)S3)c1	Z90278458	-7.82	-6.75	3-[(7S)-3-[(3-methoxyphenoxy)methyl]-7-methyl-7H-[1,2,4]triazolo[3,4-b][1,3,4]thiadiazin-6-yl]-2-methyl-1H-indole
COc1ccccc1C1=Nn2c(Cc3cccc4ccccc34)nnc2SC1	Z55659743	-7.72	-6.33	6-(2-methoxyphenyl)-3-[(naphthalen-1-yl)methyl]-5H-[1,2,4]triazolo[3,4-b][1,3,4]thiadiazine
COc1ccc(OCc2nnc3n2N=C(c2ccc4c(c2)CCO4)CS3)cc1	Z667825018	-7.70	-5.04	6-(2,3-dihydro-1-benzofuran-5-yl)-3-[(4-methoxyphenoxy)methyl]-5H-[1,2,4]triazolo[3,4-b][1,3,4]thiadiazine
COC(=O)[C]1Sc2nnc(COc3ccc(OC)cc3)n2N=C1c1ccc2c(c1)CCO2	Z8040061307	-7.64	-5.57	6-(2,3-dihydro-1-benzofuran-5-yl)-7-(methoxycarbonyl)-3-[(4-methoxyphenoxy)methyl]-7H-[1,2,4]triazolo[3,4-b][1,3,4]thiadiazin-7-yl
Cc1ccc(C2=Nn3c(COc4ccc(F)cc4)nnc3S[C@@H]2CC(=O)[O-])cc1	Z141354596	-7.56	-1.37	2-{3-[(4-fluorophenoxy)methyl]-6-(4-methylphenyl)-5H-[1,2,4]triazolo[3,4-b][1,3,4]thiadiazin-7-yl}acetic acid
CCOc1ccccc1OCc1nnc2n1N=C(c1ccc(C)c(C)c1)[C@@H](C)S2	Z141358858	-7.55	-7.16	(7R)-6-(3,4-dimethylphenyl)-3-[(2-ethoxyphenoxy)methyl]-7-methyl-7H-[1,2,4]triazolo[3,4-b][1,3,4]thiadiazine
COc1ccc(OCc2nnc3n2N=C(c2ccc(F)cc2)[C@@H](CC(=O)[O-])S3)cc1	Z90276647	-7.49	-1.29	2-[6-(4-fluorophenyl)-3-[(4-methoxyphenoxy)methyl]-5H-[1,2,4]triazolo[3,4-b][1,3,4]thiadiazin-7-yl]acetic acid
COc1ccc(OCc2nnc3n2N=C(c2cc(C)[nH]c2C)CS3)cc1	Z667825046	-7.49	-4.26	3-{3-[(4-methoxyphenoxy)methyl]-5H-[1,2,4]triazolo[3,4-b][1,3,4]thiadiazin-6-yl}-2,5-dimethyl-1H-pyrrole
COc1ccc(OCc2nnc3n2N=C(c2ccc(NC(=O)CC(C)C)cc2)CS3)cc1	Z55660047	-7.47	-6.02	N-(4-{3-[(4-methoxyphenoxy)methyl]-5H-[1,2,4]triazolo[3,4-b][1,3,4]thiadiazin-6-yl}phenyl)-3-methylbutanamide
CCOC(=O)[C@H]1Sc2nnc(COc3ccc(OC)cc3)n2N=C1c1ccc(NC(C)=O)cc1	Z8166491807	-7.46	-5.81	ethyl (7S)-6-(4-acetamidophenyl)-3-[(4-methoxyphenoxy)methyl]-7H-[1,2,4]triazolo[3,4-b][1,3,4]thiadiazine-7-carboxylate
CCOC(=O)[C-]1Sc2nnc(COc3ccc(OC)cc3)n2N=C1c1ccc([N+](=O)[O-])cc1	Z8186118910	-7.37	-5.63	ethyl 3-[(4-methoxyphenoxy)methyl]-6-(4-nitrophenyl)-5H-[1,2,4]triazolo[3,4-b][1,3,4]thiadiazine-7-carboxylate
CCOC(=O)[C@H]1Sc2nnc(COc3ccc(OC)cc3)n2N=C1c1ccc(NC(=O)CC(C)C)cc1	Z8024365455	-7.37	-6.97	ethyl (7S)-3-[(4-methoxyphenoxy)methyl]-6-[4-(3-methylbutanamido)phenyl]-7H-[1,2,4]triazolo[3,4-b][1,3,4]thiadiazine-7-carboxylate
CCOC(=O)[C@H]1Sc2nnc(COc3ccc(OC)cc3)n2N=C1c1ccc(N)cc1	Z7310720580	-7.33	-5.65	ethyl (7S)-6-(4-aminophenyl)-3-[(4-methoxyphenoxy)methyl]-7H-[1,2,4]triazolo[3,4-b][1,3,4]thiadiazine-7-carboxylate
Cc1ccc(C2=Nn3c(nnc3-c3ccco3)S[C@H]2CC(=O)[O-])cc1	Z90276386	-7.23	-1.73	2-[3-(furan-2-yl)-6-(4-methylphenyl)-5H-[1,2,4]triazolo[3,4-b][1,3,4]thiadiazin-7-yl]acetic acid
COC(=O)[C@H]1Sc2nnc(COc3ccc(OC)cc3)n2N=C1c1ccc(OC)cc1OC	Z8024373548	-7.20	-5.41	methyl (7S)-6-(2,4-dimethoxyphenyl)-3-[(4-methoxyphenoxy)methyl]-7H-[1,2,4]triazolo[3,4-b][1,3,4]thiadiazine-7-carboxylate
CC(=O)Nc1ccc(C2=Nn3c(nnc3-c3cccnc3)SC2)cc1	Z55659325	-7.12	-4.51	N-{4-[3-(pyridin-3-yl)-5H-[1,2,4]triazolo[3,4-b][1,3,4]thiadiazin-6-yl]phenyl}acetamide
COC(=O)[C]1Sc2nnc(COc3ccc(OC)cc3)n2N=C1c1cccs1	Z8024491322	-7.10	-5.36	7-(methoxycarbonyl)-3-[(4-methoxyphenoxy)methyl]-6-(thiophen-2-yl)-7H-[1,2,4]triazolo[3,4-b][1,3,4]thiadiazin-7-yl
Clc1ccc(C2=Nn3c(nnc3-c3cccnc3)SC2)cc1	Z55659297	-7.00	-5.16	3-[6-(4-chlorophenyl)-5H-[1,2,4]triazolo[3,4-b][1,3,4]thiadiazin-3-yl]pyridine
COc1ccc(OCc2nnc3n2N=C(c2cccs2)CS3)cc1	Z55627843	-6.96	-4.81	3-[(4-methoxyphenoxy)methyl]-6-(thiophen-2-yl)-5H-[1,2,4]triazolo[3,4-b][1,3,4]thiadiazine
COc1ccc(OCc2nnc3n2N=C(c2ccc[nH]2)CS3)cc1	Z667824920	-6.93	-4.30	2-{3-[(4-methoxyphenoxy)methyl]-5H-[1,2,4]triazolo[3,4-b][1,3,4]thiadiazin-6-yl}-1H-pyrrole
CCc1nnc2n1N=C(c1ccc(C)cc1)[C@@H](CC(=O)[O-])S2	Z90278306	-6.65	-0.16	2-[3-ethyl-6-(4-methylphenyl)-5H-[1,2,4]triazolo[3,4-b][1,3,4]thiadiazin-7-yl]acetic acid
COc1ccc(C2=Nn3c(nnc3-c3ccncc3)SC2)cc1OC	Z55659418	-6.54	-4.40	4-[6-(3,4-dimethoxyphenyl)-5H-[1,2,4]triazolo[3,4-b][1,3,4]thiadiazin-3-yl]pyridine
CCOc1ccccc1-c1nnc2n1N=C(c1ccc(OC)c(OC)c1)CS2	Z90279873	-6.52	-5.84	6-(3,4-dimethoxyphenyl)-3-(2-ethoxyphenyl)-5H-[1,2,4]triazolo[3,4-b][1,3,4]thiadiazine
COc1ccccc1C1=Nn2c(nnc2-c2ccccc2OC)SC1	Z55659692	-6.41	-5.58	3,6-bis(2-methoxyphenyl)-5H-[1,2,4]triazolo[3,4-b][1,3,4]thiadiazine

* Best Vina-predicted binding affinity score (in kcal/mol) as derived by GNINA docking into pose 7 of RGS14 (PDB id 2JNU).

§ Predicted aqueous solubility at pH 7.4 (in log_{10}[mol/L]) as derived by Chemaxon's chemicalize.com webserver (9, 10).

Enamine Name	[Absorption/Human Oral Bioavailability 20%] Interpretation (Confidence)	[Absorption/Human Oral Bioavailability 50%] Interpretation (Confidence)	[Absorption/Human Intestinal Absorption] Interpretation (Confidence)	[Absorption/P-Glycoprotein Inhibitor] Interpretation (Confidence)	[Absorption/P-Glycoprotein Substrate] Interpretation (Confidence)	[Distribution/Blood-Brain Barrier] Interpretation (Confidence)	[Metabolism/CYP 1A2 Inhibitor] Interpretation (Confidence)	[Metabolism/CYP 1A2_substrate] Interpretation (Confidence)	[Metabolism/CYP 2C19 Inhibitor] Interpretation (Confidence)	[Metabolism/CYP 2C19_substrate] Interpretation (Confidence)	[Metabolism/CYP 2C9 Inhibitor] Interpretation (Confidence)	[Metabolism/CYP 2C9 Substrate] Interpretation (Confidence)	[Metabolism/CYP 2D6 Inhibitor] Interpretation (Confidence)	[Metabolism/CYP 2D6 Substrate] Interpretation (Confidence)	[Metabolism/CYP 3A4 Inhibitor] Interpretation (Confidence)	[Metabolism/CYP 3A4 Substrate] Interpretation (Confidence)	[Toxicity/AMES Mutagenesis] Interpretation (Confidence)	[Toxicity/ Carcinogenesis] Interpretation (Confidence)	[Toxicity/hERG Blockers] Interpretation (Confidence)
Z90276197	Bioavailable (Medium)	Bioavailable (Medium)	Absorbed (High)	Non-Inhibitor (High)	Non-Substrate (High)	Penetrable (High)	Inhibitor (High)	Non-Substrate (High)	Non-Inhibitor (Medium)	Non-Substrate (Low)	Inhibitor (Medium)	Non-Substrate (Medium)	Non-Inhibitor (High)	Non-Substrate (Low)	Non-Inhibitor (Medium)	Non-Substrate (Medium)	Safe (High)	Safe (Medium)	Safe (High)
Z90275816	Bioavailable (Low)	Bioavailable (Medium)	Absorbed (High)	Non-Inhibitor (High)	Non-Substrate (High)	Penetrable (High)	Inhibitor (High)	Non-Substrate (High)	Inhibitor (Low)	Non-Substrate (Low)	Inhibitor (Medium)	Non-Substrate (Medium)	Non-Inhibitor (High)	Non-Substrate (Medium)	Inhibitor (Medium)	Non-Substrate (High)	Toxic (Low)	Safe (High)	Safe (Low)
Z90277093	Bioavailable (Low)	Bioavailable (Medium)	Absorbed (High)	Non-Inhibitor (High)	Non-Substrate (High)	Non-Penetrable (Medium)	Non-Inhibitor (High)	Non-Substrate (High)	Non-Inhibitor (High)	Non-Substrate (Medium)	Non-Inhibitor (Low)	Non-Substrate (High)	Non-Inhibitor (High)	Non-Substrate (High)	Non-Inhibitor (Medium)	Non-Substrate (High)	Safe (High)	Safe (Low)	Safe (High)
Z90276771	Bioavailable (Medium)	Bioavailable (Medium)	Absorbed (High)	Non-Inhibitor (High)	Non-Substrate (High)	Penetrable (High)	Inhibitor (High)	Non-Substrate (High)	Non-Inhibitor (Medium)	Non-Substrate (Low)	Non-Inhibitor (Low)	Non-Substrate (Medium)	Non-Inhibitor (Medium)	Non-Substrate (Medium)	Non-Inhibitor (Medium)	Non-Substrate (Medium)	Safe (High)	Safe (Medium)	Safe (High)
Z90277285	Bioavailable (Medium)	Bioavailable (Low)	Absorbed (High)	Non-Inhibitor (High)	Non-Substrate (High)	Penetrable (High)	Inhibitor (Medium)	Non-Substrate (High)	Non-Inhibitor (High)	Non-Substrate (Low)	Non-Inhibitor (Medium)	Non-Substrate (Low)	Non-Inhibitor (High)	Non-Substrate (Low)	Non-Inhibitor (Low)	Non-Substrate (High)	Safe (High)	Safe (High)	Safe (High)
Z90275304	Bioavailable (Medium)	Bioavailable (Medium)	Absorbed (High)	Non-Inhibitor (High)	Non-Substrate (High)	Penetrable (High)	Non-Inhibitor (Low)	Non-Substrate (High)	Non-Inhibitor (Medium)	Non-Substrate (Medium)	Non-Inhibitor (Medium)	Non-Substrate (High)	Non-Inhibitor (High)	Non-Substrate (High)	Non-Inhibitor (High)	Non-Substrate (High)	Safe (Low)	Safe (Low)	Safe (Low)
Z90278051	Bioavailable (Medium)	Bioavailable (Low)	Absorbed (High)	Non-Inhibitor (High)	Non-Substrate (High)	Penetrable (Medium)	Inhibitor (High)	Non-Substrate (Medium)	Non-Inhibitor (Low)	Non-Substrate (Low)	Non-Inhibitor (High)	Non-Substrate (High)	Non-Inhibitor (High)	Non-Substrate (Low)	Non-Inhibitor (High)	Non-Substrate (Low)	Safe (Medium)	Safe (High)	Safe (High)
Z90276194	Bioavailable (Medium)	Bioavailable (Medium)	Absorbed (High)	Non-Inhibitor (High)	Non-Substrate (High)	Penetrable (Low)	Inhibitor (Medium)	Non-Substrate (High)	Inhibitor (Low)	Non-Substrate (Medium)	Inhibitor (Medium)	Non-Substrate (High)	Non-Inhibitor (High)	Non-Substrate (High)	Non-Inhibitor (High)	Substrate (Low)	Safe (High)	Safe (Low)	Safe (Medium)
Z90278242	Bioavailable (Medium)	Bioavailable (Medium)	Absorbed (High)	Non-Inhibitor (High)	Non-Substrate (High)	Penetrable (High)	Non-Inhibitor (Low)	Non-Substrate (High)	Inhibitor (Low)	Non-Substrate (Medium)	Non-Inhibitor (Low)	Non-Substrate (High)	Non-Inhibitor (High)	Non-Substrate (Medium)	Non-Inhibitor (High)	Non-Substrate (Low)	Safe (High)	Toxic (Low)	Safe (High)
Z90276389	Bioavailable (Medium)	Bioavailable (Low)	Absorbed (High)	Non-Inhibitor (High)	Non-Substrate (High)	Penetrable (High)	Inhibitor (High)	Non-Substrate (Medium)	Non-Inhibitor (Low)	Non-Substrate (Medium)	Non-Inhibitor (Medium)	Non-Substrate (High)	Non-Inhibitor (High)	Non-Substrate (Low)	Non-Inhibitor (High)	Non-Substrate (High)	Safe (High)	Toxic (Low)	Safe (High)
Z90278248	Bioavailable (Medium)	Bioavailable (Medium)	Absorbed (High)	Non-Inhibitor (High)	Non-Substrate (High)	Penetrable (High)	Inhibitor (High)	Non-Substrate (High)	Non-Inhibitor (Medium)	Non-Substrate (Low)	Inhibitor (Low)	Non-Substrate (Low)	Inhibitor (High)	Non-Substrate (Medium)	Non-Inhibitor (Low)	Non-Substrate (High)	Safe (High)	Safe (Medium)	Safe (High)
Z90274599	Bioavailable (Medium)	Bioavailable (Medium)	Absorbed (High)	Non-Inhibitor (High)	Non-Substrate (High)	Penetrable (High)	Inhibitor (High)	Non-Substrate (Medium)	Non-Inhibitor (Medium)	Non-Substrate (Low)	Non-Inhibitor (High)	Non-Substrate (Medium)	Non-Inhibitor (High)	Non-Substrate (Medium)	Non-Inhibitor (High)	Non-Substrate (High)	Safe (High)	Safe (High)	Safe (High)
Z90274338	Bioavailable (Medium)	Bioavailable (Medium)	Absorbed (High)	Non-Inhibitor (High)	Non-Substrate (High)	Non-Penetrable (High)	Inhibitor (Low)	Non-Substrate (High)	Non-Inhibitor (Medium)	Non-Substrate (Medium)	Inhibitor (High)	Non-Substrate (High)	Non-Inhibitor (High)	Non-Substrate (High)	Non-Inhibitor (Medium)	Non-Substrate (High)	Safe (High)	Safe (Medium)	Toxic (High)
Z90276391	Bioavailable (High)	Bioavailable (Low)	Absorbed (High)	Non-Inhibitor (High)	Non-Substrate (High)	Penetrable (High)	Inhibitor (High)	Non-Substrate (Medium)	Inhibitor (Low)	Non-Substrate (Medium)	Non-Inhibitor (Medium)	Non-Substrate (High)	Non-Inhibitor (High)	Non-Substrate (Medium)	Non-Inhibitor (High)	Non-Substrate (High)	Safe (High)	Safe (Low)	Safe (High)
Z90275560	Bioavailable (Low)	Bioavailable (Low)	Absorbed (High)	Non-Inhibitor (High)	Non-Substrate (High)	Penetrable (Low)	Non-Inhibitor (Low)	Non-Substrate (High)	Non-Inhibitor (Medium)	Non-Substrate (Medium)	Non-Inhibitor (Medium)	Non-Substrate (High)	Non-Inhibitor (High)	Non-Substrate (High)	Non-Inhibitor (High)	Non-Substrate (High)	Safe (Low)	Safe (Low)	Safe (Medium)
Z90278245	Bioavailable (Medium)	Bioavailable (Medium)	Absorbed (High)	Non-Inhibitor (High)	Non-Substrate (High)	Penetrable (High)	Inhibitor (High)	Non-Substrate (High)	Non-Inhibitor (Medium)	Non-Substrate (Low)	Non-Inhibitor (Low)	Non-Substrate (Medium)	Non-Inhibitor (High)	Non-Substrate (Medium)	Non-Inhibitor (Medium)	Non-Substrate (High)	Safe (High)	Safe (Medium)	Safe (High)
Z90275299	Bioavailable (Medium)	Bioavailable (Medium)	Absorbed (High)	Non-Inhibitor (High)	Non-Substrate (High)	Penetrable (Medium)	Inhibitor (Low)	Non-Substrate (High)	Non-Inhibitor (Low)	Non-Substrate (High)	Non-Inhibitor (Low)	Non-Substrate (High)	Non-Inhibitor (High)	Non-Substrate (High)	Non-Inhibitor (High)	Non-Substrate (High)	Safe (High)	Safe (Low)	Safe (Low)
Z90275301	Bioavailable (Medium)	Bioavailable (Low)	Absorbed (High)	Non-Inhibitor (High)	Non-Substrate (High)	Penetrable (Medium)	Non-Inhibitor (Low)	Non-Substrate (High)	Non-Inhibitor (Medium)	Non-Substrate (High)	Non-Inhibitor (Medium)	Non-Substrate (Medium)	Non-Inhibitor (High)	Non-Substrate (Medium)	Non-Inhibitor (High)	Non-Substrate (High)	Safe (High)	Safe (Low)	Safe (Medium)
Z90278437	Bioavailable (Low)	Bioavailable (Medium)	Absorbed (High)	Non-Inhibitor (High)	Non-Substrate (High)	Penetrable (Low)	Inhibitor (Medium)	Non-Substrate (High)	Non-Inhibitor (High)	Non-Substrate (Medium)	Inhibitor (Low)	Non-Substrate (High)	Non-Inhibitor (High)	Non-Substrate (High)	Non-Inhibitor (High)	Non-Substrate (High)	Safe (High)	Safe (Low)	Safe (Medium)
Z90274594	Bioavailable (Medium)	Bioavailable (Medium)	Absorbed (High)	Non-Inhibitor (High)	Non-Substrate (High)	Penetrable (Low)	Inhibitor (Low)	Non-Substrate (High)	Non-Inhibitor (High)	Non-Substrate (Medium)	Non-Inhibitor (Medium)	Non-Substrate (High)	Non-Inhibitor (High)	Non-Substrate (High)	Non-Inhibitor (High)	Non-Substrate (Medium)	Safe (Medium)	Safe (Low)	Safe (High)
Z90274597	Bioavailable (Medium)	Bioavailable (Low)	Absorbed (High)	Non-Inhibitor (High)	Non-Substrate (High)	Penetrable (High)	Inhibitor (High)	Non-Substrate (Medium)	Non-Inhibitor (High)	Non-Substrate (Low)	Non-Inhibitor (High)	Non-Substrate (High)	Non-Inhibitor (High)	Non-Substrate (Low)	Non-Inhibitor (High)	Non-Substrate (High)	Safe (High)	Safe (Medium)	Safe (High)
Z55660043	Bioavailable (Medium)	Bioavailable (Medium)	Absorbed (High)	Non-Inhibitor (High)	Non-Substrate (Medium)	Penetrable (High)	Inhibitor (High)	Substrate (Low)	Inhibitor (High)	Non-Substrate (Low)	Inhibitor (High)	Non-Substrate (Medium)	Non-Inhibitor (High)	Non-Substrate (Low)	Inhibitor (High)	Substrate (Low)	Safe (High)	Safe (High)	Safe (Medium)
Z55659515	Bioavailable (Medium)	Bioavailable (Medium)	Absorbed (High)	Inhibitor (High)	Non-Substrate (Medium)	Penetrable (High)	Inhibitor (High)	Substrate (Low)	Inhibitor (High)	Substrate (Low)	Inhibitor (High)	Non-Substrate (High)	Non-Inhibitor (High)	Non-Substrate (Low)	Inhibitor (Medium)	Substrate (Medium)	Safe (High)	Safe (Low)	Safe (High)
Z55628018	Bioavailable (Medium)	Bioavailable (Low)	Absorbed (High)	Inhibitor (High)	Non-Substrate (High)	Penetrable (High)	Inhibitor (High)	Substrate (Medium)	Inhibitor (High)	Non-Substrate (Low)	Inhibitor (High)	Non-Substrate (High)	Non-Inhibitor (High)	Non-Substrate (Low)	Inhibitor (Medium)	Non-Substrate (High)	Toxic (Medium)	Toxic (Low)	Toxic (Medium)
Z55660462	Bioavailable (Medium)	Bioavailable (Medium)	Absorbed (High)	Inhibitor (High)	Non-Substrate (High)	Penetrable (High)	Inhibitor (High)	Non-Substrate (Low)	Inhibitor (High)	Non-Substrate (Low)	Inhibitor (High)	Non-Substrate (Low)	Non-Inhibitor (Medium)	Non-Substrate (Low)	Inhibitor (Medium)	Substrate (Medium)	Safe (High)	Safe (Low)	Safe (Medium)
Z56944652	Bioavailable (Medium)	Bioavailable (Medium)	Absorbed (High)	Non-Inhibitor (High)	Non-Substrate (High)	Penetrable (High)	Inhibitor (High)	Substrate (Low)	Inhibitor (High)	Non-Substrate (Low)	Inhibitor (High)	Non-Substrate (High)	Non-Inhibitor (High)	Non-Substrate (Low)	Inhibitor (Medium)	Non-Substrate (Low)	Safe (Low)	Safe (Medium)	Safe (High)
Z55660304	Bioavailable (Medium)	Bioavailable (Medium)	Absorbed (High)	Non-Inhibitor (Medium)	Non-Substrate (Medium)	Penetrable (High)	Inhibitor (High)	Substrate (Low)	Inhibitor (High)	Non-Substrate (Low)	Inhibitor (High)	Non-Substrate (Medium)	Non-Inhibitor (High)	Non-Substrate (Low)	Inhibitor (High)	Substrate (Medium)	Toxic (Medium)	Safe (Low)	Toxic (High)
Z55627844	Bioavailable (Medium)	Bioavailable (Medium)	Absorbed (High)	Inhibitor (High)	Non-Substrate (High)	Penetrable (High)	Inhibitor (High)	Substrate (Medium)	Inhibitor (High)	Non-Substrate (Low)	Inhibitor (High)	Non-Substrate (High)	Non-Inhibitor (High)	Non-Substrate (Low)	Inhibitor (High)	Non-Substrate (High)	Safe (Low)	Safe (Low)	Safe (High)
Z56840012	Bioavailable (High)	Bioavailable (Medium)	Absorbed (High)	Non-Inhibitor (High)	Non-Substrate (High)	Penetrable (High)	Inhibitor (High)	Substrate (Low)	Inhibitor (High)	Non-Substrate (Low)	Inhibitor (High)	Substrate (Medium)	Non-Inhibitor (High)	Non-Substrate (Low)	Non-Inhibitor (Low)	Substrate (Low)	Safe (High)	Safe (Medium)	Toxic (Medium)
Z55660110	Bioavailable (Medium)	Bioavailable (Low)	Absorbed (High)	Inhibitor (High)	Non-Substrate (High)	Penetrable (High)	Inhibitor (High)	Substrate (Low)	Inhibitor (High)	Non-Substrate (Low)	Inhibitor (High)	Non-Substrate (High)	Non-Inhibitor (Medium)	Substrate (Low)	Non-Inhibitor (Medium)	Non-Substrate (High)	Safe (High)	Toxic (Low)	Safe (Low)
Z103673844	Bioavailable (Medium)	Bioavailable (Medium)	Absorbed (High)	Non-Inhibitor (High)	Non-Substrate (Medium)	Penetrable (High)	Inhibitor (High)	Substrate (Low)	Inhibitor (High)	Non-Substrate (Low)	Inhibitor (High)	Non-Substrate (Medium)	Non-Inhibitor (High)	Non-Substrate (Low)	Inhibitor (High)	Substrate (Low)	Safe (High)	Safe (High)	Safe (Medium)
Z55660524	Bioavailable (High)	Bioavailable (Medium)	Absorbed (High)	Inhibitor (High)	Non-Substrate (High)	Penetrable (High)	Inhibitor (High)	Substrate (Low)	Inhibitor (High)	Substrate (Low)	Inhibitor (High)	Non-Substrate (High)	Non-Inhibitor (High)	Substrate (Low)	Inhibitor (High)	Substrate (High)	Safe (High)	Safe (Low)	Safe (High)
Z55627782	Bioavailable (Medium)	Bioavailable (Low)	Absorbed (High)	Inhibitor (Medium)	Non-Substrate (High)	Penetrable (High)	Inhibitor (High)	Substrate (Medium)	Inhibitor (High)	Non-Substrate (Low)	Inhibitor (High)	Non-Substrate (High)	Non-Inhibitor (High)	Non-Substrate (Low)	Non-Inhibitor (High)	Non-Substrate (High)	Safe (Low)	Toxic (Low)	Safe (High)
Z55660496	Bioavailable (High)	Bioavailable (Medium)	Absorbed (High)	Non-Inhibitor (High)	Non-Substrate (High)	Penetrable (High)	Inhibitor (High)	Substrate (Low)	Inhibitor (High)	Non-Substrate (Low)	Inhibitor (High)	Non-Substrate (Medium)	Non-Inhibitor (Low)	Non-Substrate (Low)	Inhibitor (High)	Substrate (Low)	Safe (High)	Safe (Medium)	Safe (Medium)
Z667824954	Bioavailable (Medium)	Bioavailable (Medium)	Absorbed (High)	Inhibitor (High)	Non-Substrate (Medium)	Penetrable (High)	Inhibitor (High)	Non-Substrate (Medium)	Inhibitor (High)	Non-Substrate (Low)	Inhibitor (High)	Non-Substrate (High)	Non-Inhibitor (High)	Non-Substrate (Low)	Inhibitor (High)	Substrate (Medium)	Safe (Medium)	Safe (Medium)	Toxic (Low)
Z55660438	Bioavailable (High)	Bioavailable (High)	Absorbed (High)	Inhibitor (High)	Non-Substrate (High)	Penetrable (High)	Inhibitor (High)	Non-Substrate (Low)	Inhibitor (High)	Non-Substrate (Low)	Inhibitor (High)	Non-Substrate (Low)	Non-Inhibitor (High)	Non-Substrate (Low)	Inhibitor (Low)	Substrate (Medium)	Safe (High)	Safe (Low)	Safe (High)
Z667824974	Bioavailable (Medium)	Bioavailable (Medium)	Absorbed (High)	Inhibitor (High)	Non-Substrate (Medium)	Penetrable (High)	Inhibitor (High)	Non-Substrate (Low)	Inhibitor (High)	Non-Substrate (Low)	Inhibitor (High)	Non-Substrate (High)	Non-Inhibitor (High)	Non-Substrate (Low)	Inhibitor (High)	Substrate (High)	Safe (High)	Safe (Medium)	Safe (Medium)
Z55660046	Bioavailable (Medium)	Bioavailable (Medium)	Absorbed (High)	Inhibitor (High)	Non-Substrate (High)	Penetrable (High)	Inhibitor (High)	Non-Substrate (Medium)	Inhibitor (Medium)	Non-Substrate (Low)	Inhibitor (High)	Non-Substrate (Medium)	Non-Inhibitor (High)	Non-Substrate (Low)	Inhibitor (High)	Substrate (Medium)	Safe (High)	Safe (Medium)	Safe (Low)
Z7976707565	Bioavailable (Low)	Bioavailable (Medium)	Absorbed (High)	Inhibitor (High)	Non-Substrate (Medium)	Non-Penetrable (High)	Inhibitor (High)	Non-Substrate (Medium)	Inhibitor (Medium)	Non-Substrate (Low)	Inhibitor (High)	Non-Substrate (High)	Non-Inhibitor (High)	Non-Substrate (Medium)	Inhibitor (High)	Substrate (High)	Safe (High)	Safe (High)	Safe (Medium)
Z8201263931	Bioavailable (Medium)	Bioavailable (Medium)	Absorbed (High)	Inhibitor (High)	Non-Substrate (High)	Non-Penetrable (High)	Inhibitor (High)	Non-Substrate (Low)	Inhibitor (High)	Non-Substrate (Medium)	Inhibitor (High)	Non-Substrate (Medium)	Non-Inhibitor (High)	Non-Substrate (Medium)	Inhibitor (Medium)	Non-Substrate (High)	Safe (High)	Safe (High)	Toxic (High)
Z55660052	Bioavailable (Medium)	Bioavailable (Medium)	Absorbed (High)	Inhibitor (Medium)	Non-Substrate (High)	Penetrable (High)	Inhibitor (High)	Non-Substrate (Low)	Inhibitor (High)	Non-Substrate (Low)	Inhibitor (High)	Non-Substrate (High)	Non-Inhibitor (High)	Non-Substrate (Low)	Inhibitor (High)	Substrate (Low)	Safe (High)	Safe (Medium)	Toxic (Medium)
Z55660592	Bioavailable (High)	Bioavailable (High)	Absorbed (High)	Inhibitor (Low)	Non-Substrate (High)	Penetrable (High)	Inhibitor (High)	Substrate (Low)	Inhibitor (High)	Substrate (Low)	Inhibitor (High)	Non-Substrate (High)	Non-Inhibitor (High)	Non-Substrate (Low)	Inhibitor (Low)	Substrate (Medium)	Safe (High)	Safe (Medium)	Toxic (Low)
Z90277200	Bioavailable (Medium)	Bioavailable (Medium)	Absorbed (High)	Inhibitor (High)	Non-Substrate (Medium)	Penetrable (High)	Non-Inhibitor (Low)	Non-Substrate (Low)	Inhibitor (High)	Non-Substrate (Low)	Inhibitor (High)	Non-Substrate (Low)	Non-Inhibitor (High)	Non-Substrate (Low)	Inhibitor (Medium)	Substrate (Medium)	Safe (High)	Safe (Medium)	Toxic (High)
Z90276813	Bioavailable (Medium)	Bioavailable (Medium)	Absorbed (High)	Inhibitor (High)	Non-Substrate (High)	Penetrable (High)	Inhibitor (Medium)	Non-Substrate (Medium)	Inhibitor (High)	Non-Substrate (Low)	Inhibitor (High)	Non-Substrate (High)	Inhibitor (Medium)	Non-Substrate (Low)	Inhibitor (Medium)	Substrate (High)	Safe (High)	Safe (Medium)	Toxic (Medium)
Z55660805	Bioavailable (Medium)	Bioavailable (Medium)	Absorbed (High)	Inhibitor (High)	Non-Substrate (Medium)	Penetrable (High)	Inhibitor (High)	Substrate (Low)	Inhibitor (High)	Substrate (Low)	Inhibitor (High)	Non-Substrate (Medium)	Non-Inhibitor (High)	Substrate (Low)	Inhibitor (High)	Substrate (High)	Safe (High)	Safe (Medium)	Safe (Medium)
Z55660788	Bioavailable (Medium)	Bioavailable (Medium)	Absorbed (High)	Inhibitor (Medium)	Non-Substrate (Medium)	Penetrable (High)	Inhibitor (High)	Substrate (Low)	Inhibitor (High)	Non-Substrate (Low)	Inhibitor (High)	Non-Substrate (Medium)	Non-Inhibitor (High)	Substrate (Low)	Inhibitor (High)	Substrate (Low)	Safe (High)	Safe (Medium)	Safe (Low)
Z90278674	Bioavailable (Medium)	Bioavailable (Low)	Absorbed (High)	Inhibitor (High)	Non-Substrate (Medium)	Penetrable (High)	Non-Inhibitor (Low)	Non-Substrate (Low)	Inhibitor (High)	Substrate (Low)	Inhibitor (High)	Non-Substrate (Medium)	Non-Inhibitor (High)	Non-Substrate (Low)	Inhibitor (High)	Substrate (Medium)	Safe (Medium)	Safe (Medium)	Toxic (Medium)
Z90277842	Bioavailable (Medium)	Bioavailable (Medium)	Absorbed (High)	Non-Inhibitor (High)	Non-Substrate (High)	Penetrable (High)	Inhibitor (High)	Non-Substrate (Low)	Inhibitor (Medium)	Substrate (Low)	Inhibitor (High)	Non-Substrate (Medium)	Non-Inhibitor (High)	Non-Substrate (Low)	Inhibitor (Low)	Substrate (Low)	Safe (High)	Safe (Low)	Safe (High)
Z90278035	Bioavailable (Medium)	Bioavailable (Medium)	Absorbed (High)	Non-Inhibitor (High)	Non-Substrate (High)	Penetrable (High)	Inhibitor (High)	Non-Substrate (Low)	Non-Inhibitor (High)	Substrate (Low)	Non-Inhibitor (High)	Substrate (Medium)	Non-Inhibitor (High)	Substrate (Low)	Non-Inhibitor (High)	Substrate (Low)	Safe (High)	Safe (High)	Safe (High)

Supplementary Table S3. Predicted pharmacokinetic and toxicity profiles of triazolothiadiazine analogs using the Deep-PK deep-learning platform. Pharmacokinetic and toxicity properties of the triazolothiadiazine analogs were computationally predicted using the Deep-PK platform (11), a graph neural network-based tool trained on curated experimental datasets encompassing 73 ADMET-related endpoints. Each row lists a triazolothiadiazine compound previously characterized in GAP inhibition assays, with the compound name color-coded as in prior figures (e.g., red background denotes the presence of a carboxylic acid R_3-group). The table columns reflect Deep-PK predictions across key ADMET categories: absorption (including oral bioavailability and intestinal uptake), distribution (e.g., blood–brain barrier penetration), metabolism (inhibition and substrate status for major cytochrome P450 isoforms), and toxicity (mutagenicity, carcinogenicity, and hERG blockade). Each cell includes both a qualitative classification (e.g., “Bioavailable”, “Safe”, “Non-Inhibitor”) and a parenthetical confidence score assigned by the model. Background shading of table cells provides interpretative cues: green cells denote “favorable” or “safe” predictions with medium to high confidence, whereas yellow cells indicate predictions that may warrant caution, reflecting borderline confidence levels, potentially concerning classifications (*e.g.*, weak CYP inhibition), or lower prediction certainty.

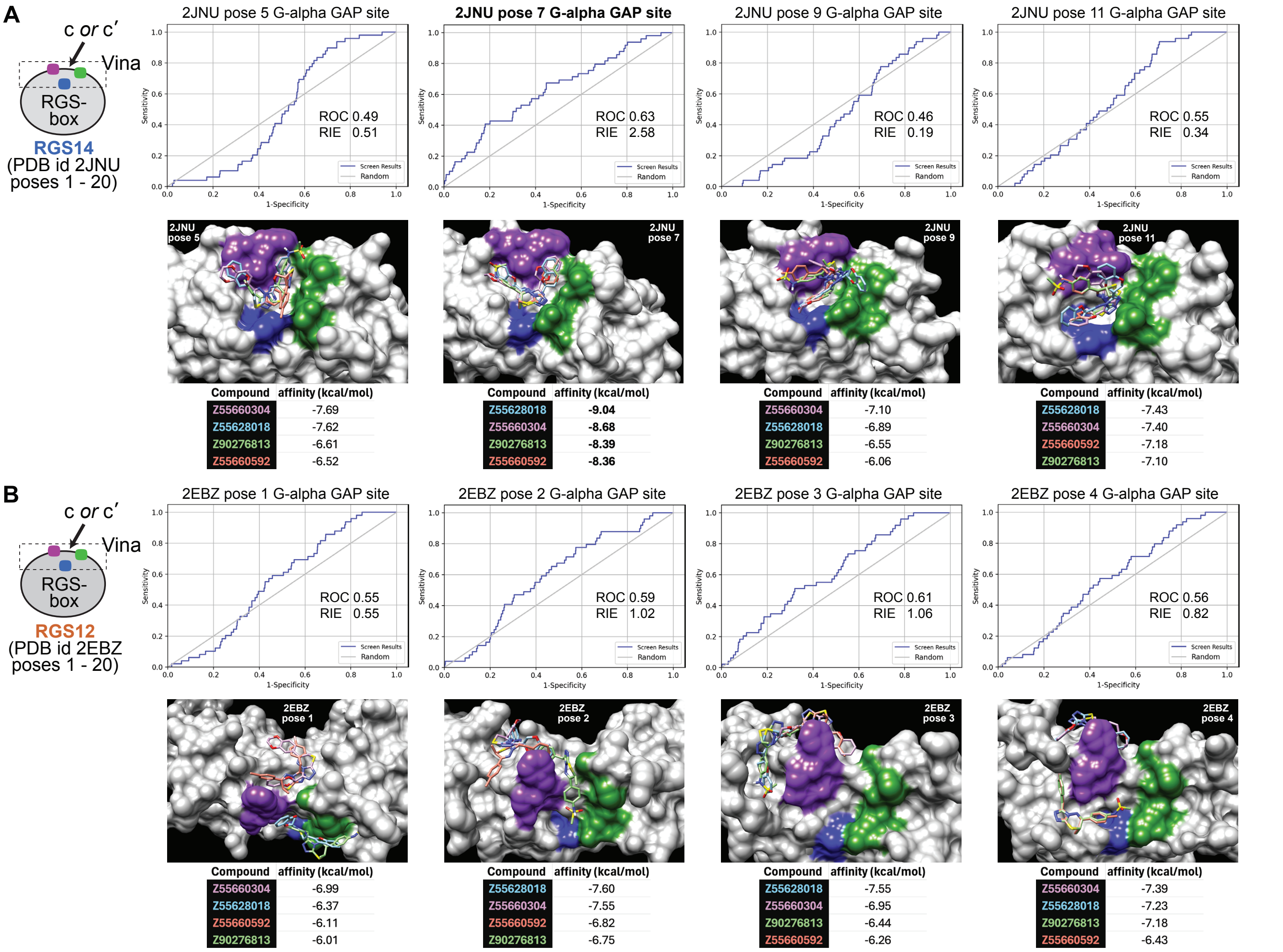

Supplementary Figure S9. Enrichment of active triazolothiadiazine ligands into the Gα-binding site across multiple RGS-box conformers. (**A-B**) Structure-based virtual screening results for selected conformers of the RGS14 (PDB id 2JNU; panel A) and RGS12 (PDB id 2EBZ; panel B) RGS-box domains, each assessed using AutoDock Vina (v1.2.5), for their ability to discriminate between active and decoy ligands. Ligand sets comprised either the 49 active triazolothiadiazine compounds (denoted as "compound" or "c") or 4,160 physicochemically matched decoy structures generated by DUD-E (denoted as "compound-prime" or c′). Each receptor conformer is labeled with its pose number and the location of the presumptive Gα-binding groove (color-coded as in Figure 2). ROC AUC and robust initial enrichment (RIE) values are shown for each pose, reflecting the degree to which active compounds were preferentially ranked within the top of the docking score distribution. Pose 7 of 2JNU yielded the highest enrichment (ROC AUC = 0.63, RIE = 2.58).

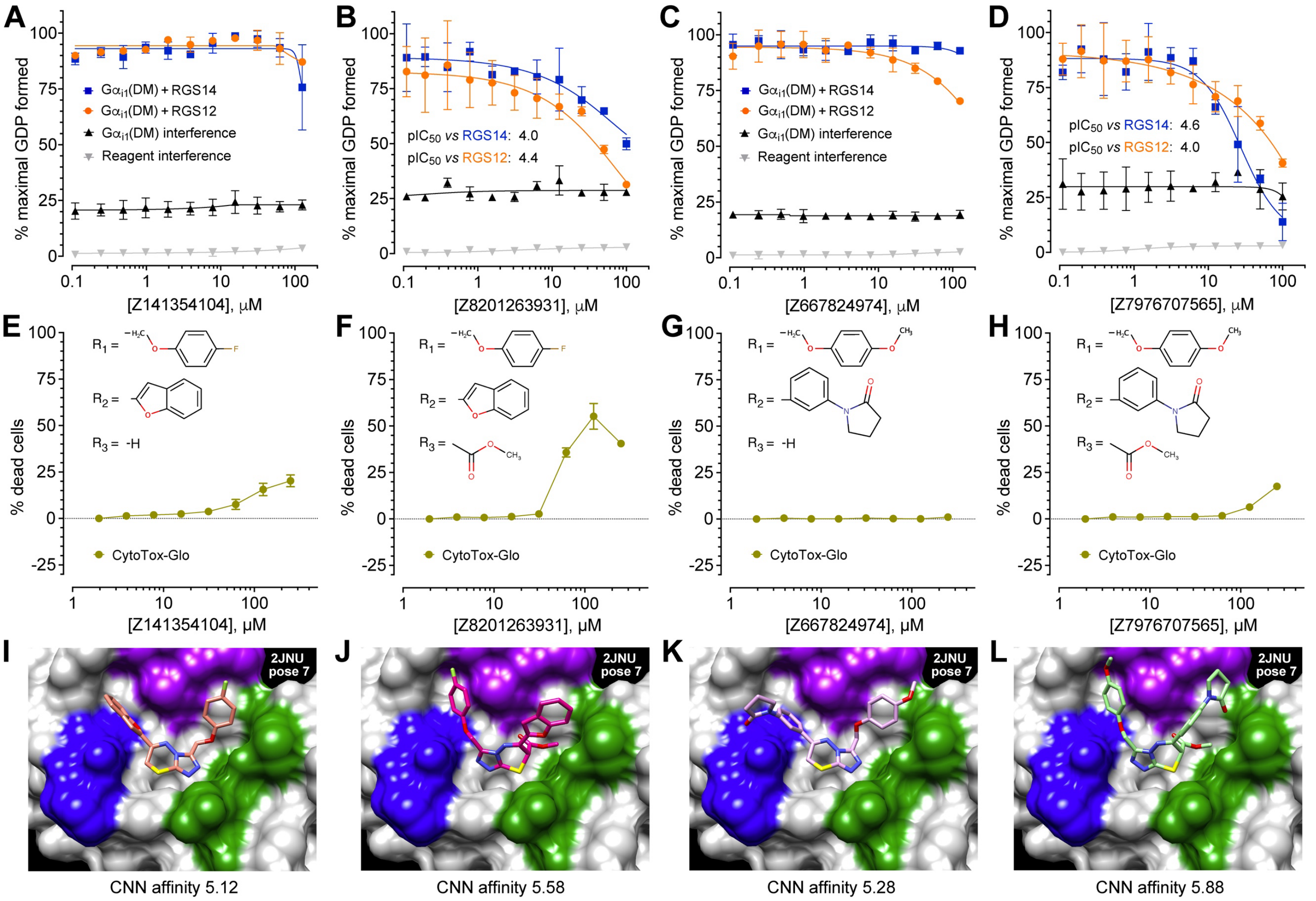

Supplementary Figure S10. Inhibitors of the triazolothiadiazine chemotype increase in potency against RGS14 GAP activity, but also in cytotoxicity, upon provision of a methyl ester R_3-group. (**A-D**) Transcreener® GDP-detection RGScreen™ assay measuring GDP production as a function of compound concentration for representative members of the active triazolothiadiazine series. GDP production was catalyzed by $G\alpha_{i1}$(DM) protein and accelerated by the indicated RGS-box protein. Additional controls demonstrate lack of signal interference by the tested compound alone (*i.e.*, on detection reagents) or intrinsic $G\alpha_{i1}$(DM) GTPase activity. (**E–H**) Cytotoxicity profiles for the same compounds in THP1-Dual™ cells using the CytoTox-Glo assay (Promega). Compounds were added in a 2-fold dilution series beginning at 250 µM and incubated overnight. Luminescent signal corresponding to dead-cell protease activity was measured first, followed by digitonin lysis and total-cell signal acquisition. Percent cytotoxicity was calculated per the manufacturer's instructions. (**I–L**) GNINA docking results for Z141354104 (panel I) and related congener Z8201263931 (panel J), and for Z667824974 (panel K) and related congener Z7976707565 (panel L), into pose 7 of the RGS14 RGS-box (PDB id 2JNU). Each pair represents a switch from lacking a formal R3-group (left) to a methyl ester (right), resulting in increased CNN affinity scores (*e.g.*, from 5.12 to 5.58 for panels I *vs* J) and a reciprocal "flip" in predicted binding orientation, whereby R1 and R2 substituents project into opposite faces of the shallow canyon at the presumptive Gα-binding interface. We are defining this predicted flipped orientation binding behavior as "ambidextrous" binding.

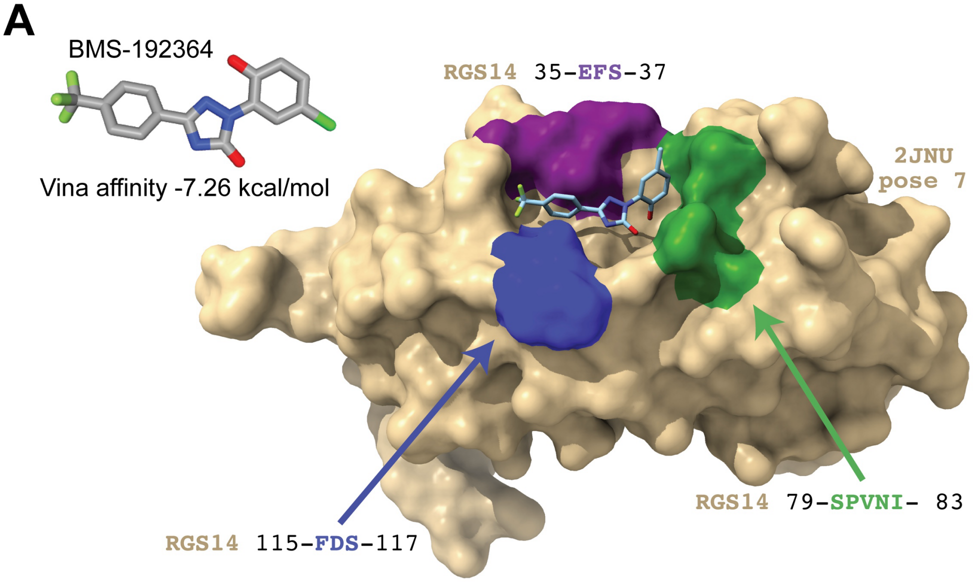

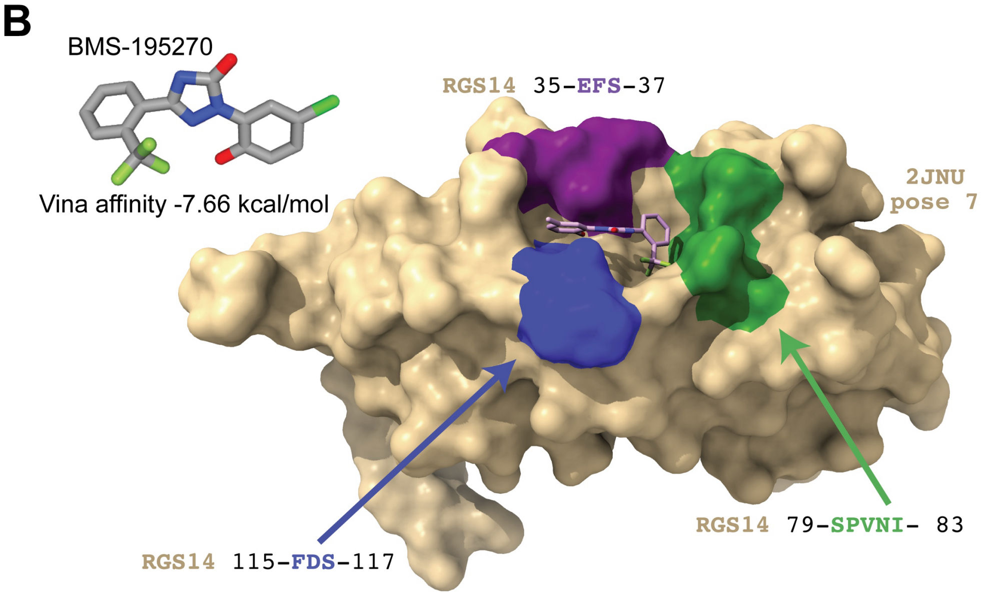

Supplementary Figure S11. Comparison of predicted binding modes for original BMS diaryl-triazolone inhibitors docked into the presumptive Gα-binding groove of RGS14. (A) Predicted pose of BMS-192364 (Vina affinity score: –7.26 kcal/mol) docked into the shallow, solvent-exposed canyon at the presumptive Gα-binding interface of pose 7 of the RGS14 RGS-box (PDB id 2JNU), as identified by GNINA-based global docking. **(B)** Predicted binding pose of BMS-195270 (Vina affinity score: –7.66 kcal/mol), a structurally related compound shown to be weakly active in vitro, similarly docked into the same receptor conformation. The spatial proximity of RGS14 residues E35, F36, S37, S79, P80, V81, N82, I83, F115, D116, and S117 (labels color-coded to match Figure 1 orientation markers) highlights the shallow nature of the Gα-interaction site and supports its predicted engagement by both ligands. Notably, these BMS compounds also exhibit flipped or "ambidextrous" orientations of substituent groups around their central core, consistent with the alternating R1/R2 projection pattern observed in the active triazolothiadiazine series.

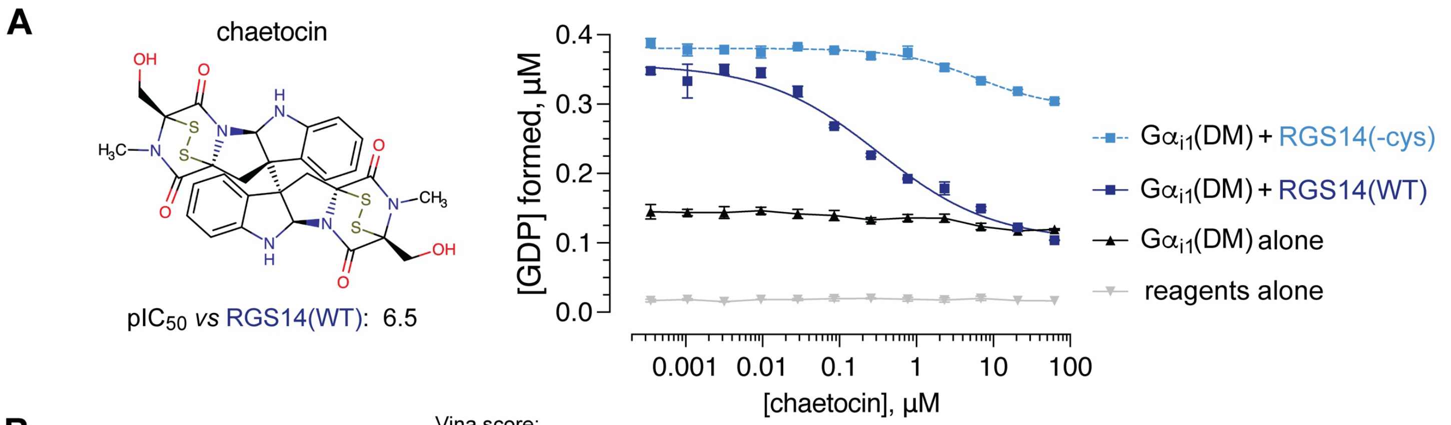

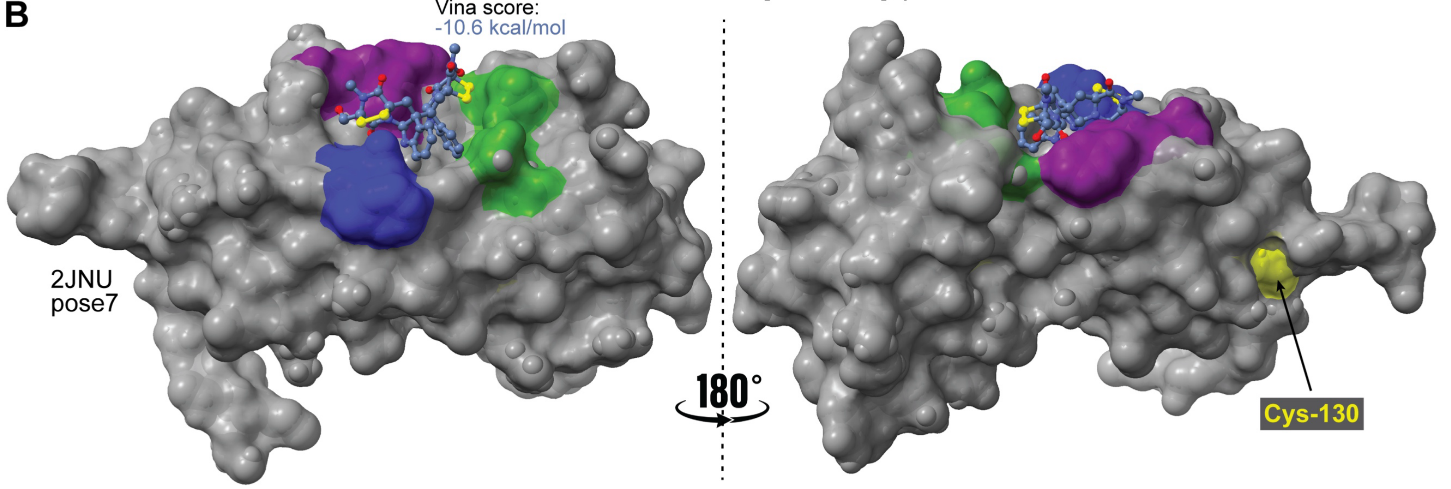

Supplementary Figure S12. Chaetocin, an inhibitor of thioredoxin reductase and histone methyltransferases, also inhibits RGS14 RGS-box GAP activity *in vitro* but predominantly via cysteine reactivity. (A) *Left,* structure of chaetocin, a 696.8 Da fungal mycotoxin identified in this study by GNINA-based *in silico* screening of the RGS14 RGS-box shallow canyon (specified by PDB id 2JNU pose 7) against MolPort's 235,298-element "Protein-Protein Interactions Library" of commercially available compounds >500 Da (acquired from molport.com on 01.03.2025). Right, Transcreener® GDP-detection RGScreen™ assay results indicating that chaetocin specifically inhibits RGS14 RGS-box protein GAP activity towards $G\alpha_{i1}$(DM) protein when wildtype RGS14 sequence protein is used ("WT"; estimated IC_{50} of 0.32 µM), and this inhibitory activity is largely lost when using a cysteine-free version of the recombinant RGS14 RGS-box protein ("-cys"), implicating cysteine modification as the predominant mechanism of (allosteric) inhibition. **(B)** Three-dimensional representation of the highest scoring docked pose of chaetocin within the Gα-interacting shallow canyon of the RGS14 RGS-box. The considerable distance between chaetocin's internal disulfide bonds (yellow) and Cys-130 of RGS14 (also yellow), located at the allosteric 'hot-spot' distal to the Gα-binding interface, suggests potential thiol-based covalent modification rather than canonical PPI groove engagement.

References for Supplementary Information section

1. Uhlen, M., Fagerberg, L., Hallstrom, B. M., Lindskog, C., Oksvold, P., Mardinoglu, A. *et al.* (2015) Proteomics. Tissue-based map of the human proteome. *Science* **347**, 1260419. 10.1126/science.1260419
2. Wu, C., Orozco, C., Boyer, J., Leglise, M., Goodale, J., Batalov, S. *et al.* (2009) BioGPS: an extensible and customizable portal for querying and organizing gene annotation resources. *Genome Biol* **10**, R130. 10.1186/gb-2009-10-11-r130
3. Costanzo, M. C., von Grotthuss, M., Massung, J., Jang, D., Caulkins, L., Koesterer, R. *et al.* (2023) The Type 2 Diabetes Knowledge Portal: An open access genetic resource dedicated to type 2 diabetes and related traits. *Cell Metab* **35**, 695-710 e696. 10.1016/j.cmet.2023.03.001
4. de Leeuw, C. A., Mooij, J. M., Heskes, T., and Posthuma, D. (2015) MAGMA: generalized gene-set analysis of GWAS data. *PLoS Comput Biol* **11**, e1004219. 10.1371/journal.pcbi.1004219
5. Zielinski, T., Kimple, A. J., Hutsell, S. Q., Koeff, M. D., Siderovski, D. P., and Lowery, R. G. (2009) Two Galpha(i1) rate-modifying mutations act in concert to allow receptor-independent, steady-state measurements of RGS protein activity. *J Biomol Screen* **14**, 1195-1206. 10.1177/1087057109347473
6. Bosch, D. E., Zielinski, T., Lowery, R. G., and Siderovski, D. P. (2012) Evaluating modulators of "Regulator of G-protein Signaling" (RGS) proteins. *Curr Protoc Pharmacol* **Chapter 2**, Unit2 8. 10.1002/0471141755.ph0208s56
7. Zhang, J. H., Chung, T. D., and Oldenburg, K. R. (1999) A Simple Statistical Parameter for Use in Evaluation and Validation of High Throughput Screening Assays. *J Biomol Screen* **4**, 67-73. 10.1177/108705719900400206
8. Atomwise, A. P. (2024) AI is a viable alternative to high throughput screening: a 318-target study. *Sci Rep* **14**, 7526. 10.1038/s41598-024-54655-z
9. Shoghi, E., Fuguet, E., Bosch, E., and Rafols, C. (2013) Solubility-pH profiles of some acidic, basic and amphoteric drugs. *Eur J Pharm Sci* **48**, 291-300. 10.1016/j.ejps.2012.10.028
10. Hou, T. J., Xia, K., Zhang, W., and Xu, X. J. (2004) ADME evaluation in drug discovery. 4. Prediction of aqueous solubility based on atom contribution approach. *J Chem Inf Comput Sci* **44**, 266-275. 10.1021/ci034184n
11. Myung, Y., de Sa, A. G. C., and Ascher, D. B. (2024) Deep-PK: deep learning for small molecule pharmacokinetic and toxicity prediction. *Nucleic Acids Res* **52**, W469-W475. 10.1093/nar/gkae254
